# Rational Design, Synthesis, and In Vitro Activity of Heterocyclic Gamma-Butyrobetaines as Potential Carnitine Acetyltransferase Inhibitors

**DOI:** 10.3390/molecules30030735

**Published:** 2025-02-06

**Authors:** Savina Stoyanova, Milen G. Bogdanov

**Affiliations:** Faculty of Chemistry and Pharmacy, Sofia University St. Kliment Ohridski, 1, Jammes Bourchier Blvd., 1164 Sofia, Bulgaria; savina_si@abv.bg

**Keywords:** carnitine acyltransferase, L-carnitine, fatty acid oxidation, enzyme inhibition

## Abstract

This study investigates heterocyclic gamma-butyrobetaine (GBB) analogs as metabolic modulators through an integrated approach involving rational design, molecular docking, synthesis, and in vitro evaluation. The compounds synthesized demonstrated promising inhibitory potential toward carnitine acetyltransferase (CAT) and presumably other enzymes within the carnitine transferase family, with IC_50_ values ranging from 2.24 to 43.6 mM. Notably, some compounds demonstrated superior activity to the reference drug Meldonium (IC_50_ = 11.39 mM). A substantial outcome of the study that might serve as a foundation for future optimization and synthesis of more potent compounds was that a bulky, hydrophobic substituent at the gamma position enhances inhibitory activity, whereas esterification and increased polarity diminish it. The most effective compound was determined to be a reversible competitive inhibitor of CAT, with a *K*_i_ value of 3.5 mM comparable to Meldonium’s *K*_i_ of 1.63 mM. These results suggest that heterocyclic GBB analogs present potential candidates for regulating metabolic processes and treating conditions including ischemic diseases, diabetes, and specific cancers.

## 1. Introduction

Carnitine acyltransferases (CTs) constitute a family of essential mammalian cellular enzymes crucial for bioenergetic processes in human and animal cells [1,2,3,4]. Their primary function pertains to the oxidation of fatty acids (FAO) within the mitochondria, which represents a main energy source alongside glucose oxidation. However, the transport of FAs from the cytosol into the mitochondria, where FAO occurs, requires a specific mechanism because FAs cannot diffuse directly into the mitochondria [5]. This transport mechanism involves at least three distinct enzymatic reactions known as the carnitine shuttle, with the CT managing the initial and rate-limiting component by facilitating the reversible transfer of fatty-acid-derived acyl groups between coenzyme A (CoA) and the amino acid L-carnitine [6,7].

The CT family consists of three distinct subsets: carnitine acetyltransferase (CAT), carnitine octanoyltransferase (COT), and carnitine palmitoyltransferase (CPT) [8,9,10,11,12]. These subsets are responsible for the translocation of FAs with varying chain lengths between cellular compartments, specifically the cytosol and the mitochondria. The CAT subset is responsible for short-chain fatty acids, such as acetate and propionate, and is localized within the mitochondria, endoplasmic reticulum, and cytoplasmic peroxisomes [12]. The COT subset is involved in processing medium-chain fatty acids, including octanoate and heptanoate, and is predominantly situated in the cytoplasmic peroxisomes. Lastly, the CPT subset manages long-chain fatty acids, such as palmitate and stearate, and is primarily located in the inner and outer mitochondrial compartments. Despite the above differences in function and localization, these subsets share significant structural similarities, and compounds proven to affect one subset are often found to be active against the others [8,13].

**Figure 1:**
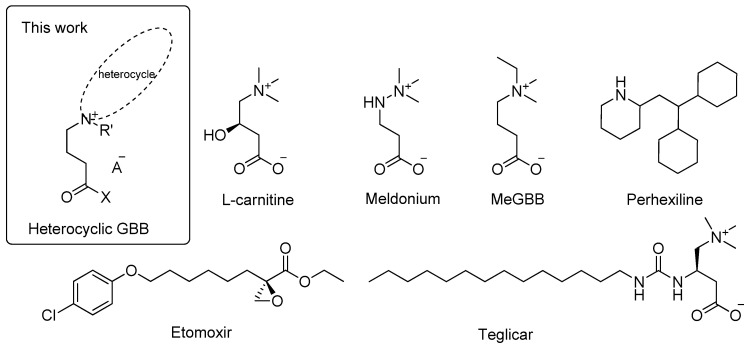
Structure of natural substrate L-carnitine, Meldonium, MeGBB, Perhexiline, Etomoxir, and Teglicar—established CT inhibitors, and general structure of compounds studied herein.

Notably, while the prevailing medical hypothesis supports the notion that increased rates of FAO are advantageous, early studies from the 1960s have contested this perspective [14]. Given these data and the crucial significance of the CT enzyme family in FAO, extensive research to elucidate their structure, function, and potential for therapeutic intervention have been undertaken over the past few decades. As a result, CT has emerged as a promising target for addressing various chronic conditions, including cardiovascular disease [15,16,17], diabetes [18,19,20,21,22,23,24], renal [25] and hepatic [26] conditions, mental [27] and neurodegenerative disorders [28,29,30], and viral pulmonary pathology [31], to name just a few. In addition, in contrast to the widely held belief that cancer cells depend on glucose for proliferation and metastasis, recent evidence has challenged this dogma by highlighting the potential role of excessive FAO in driving the cancerization of normal tissues and the metastatic process [32]. Consequently, FAO inhibition has also emerged as a highly promising therapeutic strategy for various cancer types, devoid of known resistance mechanisms characteristic of conventional cytotoxic chemotherapy [33,34,35,36,37,38,39,40,41]. All this led to the development of small-molecule compounds that function as FAO inhibitors by targeting specific subsets of the CT enzyme family, e.g., Meldonium, Etomoxir, Teglicar, and Perhexiline (Figure 1) [32].

The two most extensively researched examples of CT inhibitors are Meldonium [42,43,44] and Etomoxir [45,46]. The primary proposed mechanism of action for Meldonium (marketed as Mildronate^®^, not licensed by the FDA in the United States but produced and prescribed as a drug for treating patients with heart conditions such as angina, chronic heart failure, cardiomyopathy, and other cardiovascular disorders in several European countries) involves targeting de novo L-carnitine biosynthesis by inhibiting the enzyme gamma-butyrobetaine dioxygenase (BBOX) [47,48]. Additional research has also revealed its potential as an inhibitor of organic cation/carnitine transporter OCTN2 and CAT [44,49]. In contrast, Etomoxir (a drug candidate that failed Phase II clinical trial) primarily targets the CPT subset of enzymes while also exhibiting inhibitory activity against CAT and COT [50,51,52]. Despite their demonstrated therapeutic potential, some approved drugs (e.g., Meldonium) exhibit a number of drawbacks, including a relatively weak affinity for the CT [44], or toxicity (e.g., Etomoxir) by evoking oxidative stress, hepatoxicity, inhibition of electron transport chain complexes, etc., when used in clinically relevant concentrations [39,53,54]. Hence, the development of novel CT inhibitors with amplified potency and reduced toxicity bears considerable scientific and clinical potential [32].

Based on the above discussion, we have designed this study to demonstrate that heterocyclic gamma-butyrobetaine (GBB) analogs have the potential to serve as metabolic modulators. This potential is due to their initially hypothesized inhibitory activity against carnitine acetyltransferase and possibly other enzymes in the carnitine transferase family. Our investigation includes rational design, molecular docking, synthesis, and in vitro assay methodologies.

## 2. Results and Discussion

### 2.1. Rational Design

Understanding the active site structure and specific enzyme–substrate and enzyme–inhibitor/activator interactions is imperative in developing potent enzyme inhibitors or activators. The data presented in Table 1 [8,9,12,13,55,56], derived from isolated CATs from human, mouse, and pigeon sources [8,9,12,55,56], reveal a shared amino acid sequence in the L-carnitine binding active sites, despite variances in the polypeptide chains’ primary structures. Notably, this structural similarity extends to other enzymes within the CT family, such as COT, CPT1, and CPT2 [8,13,56]; therefore, one can confidently extrapolate the insights gained from one enzyme to the others.

**Figure 2:**
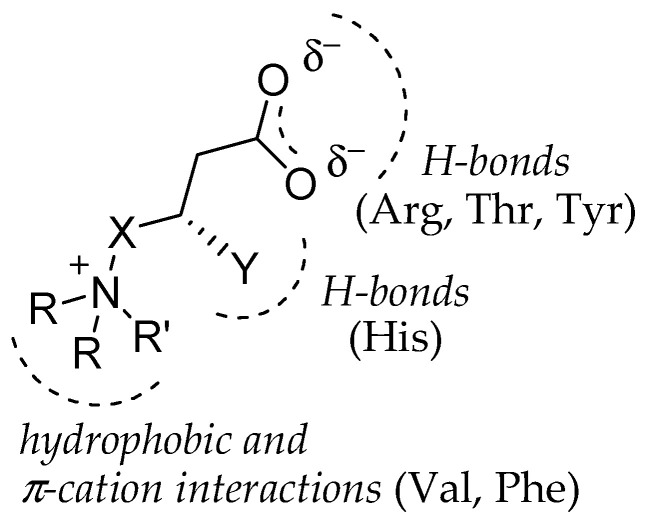
Schematic representation of deduced (see Table 1) specific interactions in enzyme–substrate complex of: L-carnitine (natural substrate): X = CH_2_; Y = OH; R = R = R′ = methyl; Meldonium (approved drug): X = NH; Y = H; R = R = R′ = methyl; MeGBB (3rd phase cl. trials): X = CH_2_; Y = H; R = R = methyl; R′ = ethyl; heterocyclic GBB: X = CH_2_; Y = H; R = R = cycle; R′ = methyl; heteroaromatic GBB: X = CH_2_; Y = H; R = R = R′ = cycle.

A range of interactions, encompassing hydrogen bonds, hydrophobic interactions, and π–cation interactions, as depicted in Figure 2, determines the efficiency and stability of the L-carnitine–CAT enzyme–substrate complex. According to the available crystallographic data [8,9,12,13,55,56], the major role is played by histidine, which dictates the acylation of the substrate—His322 for human-isolated enzymes (*Homo sapiens*, PDB identifier: 1S5O) [8] or His314 for those derived from mice (*Mus musculus*, PDB identifier: 1NDF) [9]. Specifically, the hydrogen bonding of the hydroxyl group of L-carnitine with the nitrogen atom (N3) of the imidazolium ring of histidine, followed by complete deprotonation, plays a pivotal role in shaping the stereospecificity of the reaction. In addition, the carboxyl group of the substrate forms hydrogen bonds with specific amino acids (Tyr431, Arg497, and Thr444 for human enzymes, and Tyr423, Arg489, and Thr436 for mouse-derived enzymes). Furthermore, the quaternary nitrogen atom of the substrate is positioned within a hydrophobic pocket, with its positive charge being partly stabilized by interactions with valine and phenylalanine (Val548 and Phe545 for human enzymes, and Val540 and Phe536 for mouse enzymes).

Based on the above analysis, *we hypothesized that incorporating a heterocyclic ring as a substituent in the gamma-position of butanoic acid could be a prerequisite for reinforcing the hydrophobic and π–cation interactions within the enzyme’s hydrophobic pocket* (see Figure 2). This reinforcement is expected to result in stronger inhibition of CAT when compared to Meldonium, as an example, which lacks ring substituents containing the quaternary nitrogen atom (see Figure 1). Furthermore, introducing an aromatic substituent is considered necessary to facilitate π–π interactions, further augmenting the affinity of these analogs for the enzyme’s active site.

To investigate this hypothesis, we initiated theoretical studies (molecular docking) to compare the binding affinity of hypothetical CAT inhibitors containing heterocyclic substituents with that of the natural substrate L-carnitine and the inhibitors Meldonium and 4-(ethyldimethylammonio)butanoate (MeGBB—a drug candidate in phase III of clinical trials, Figure 1). The study utilized the freely available online platform Mcule (http://mcule.com) and leveraged the structures for CAT isolated from mouse (*Mus musculus*, PDB identifier: 1NDI) [9] and CPT2 isolated from rat (*Rattus norvegicus*, PDB identifier: 2FW3) [57]. Candidate cyclic aliphatic tertiary amines including *N*-methylpyrrolidine, *N*-methylpiperidine, and *N*-methylmorpholine were chosen, along with aromatic representatives such as *N*-methylimidazole, pyridine, *N*,*N*-dimethyl-4-aminopyridine, quinoline, and isoquinoline. Additionally, the bromides of the corresponding ethyl esters of the quaternary gamma-butyrobetaines were included to evaluate the impact of hydrogen bonding. The structural features of the studied compounds and the resultant findings are concisely presented in Table 2, where a lower absolute value denotes a higher binding affinity of the inhibitor to the active site.

**Figure 3:**
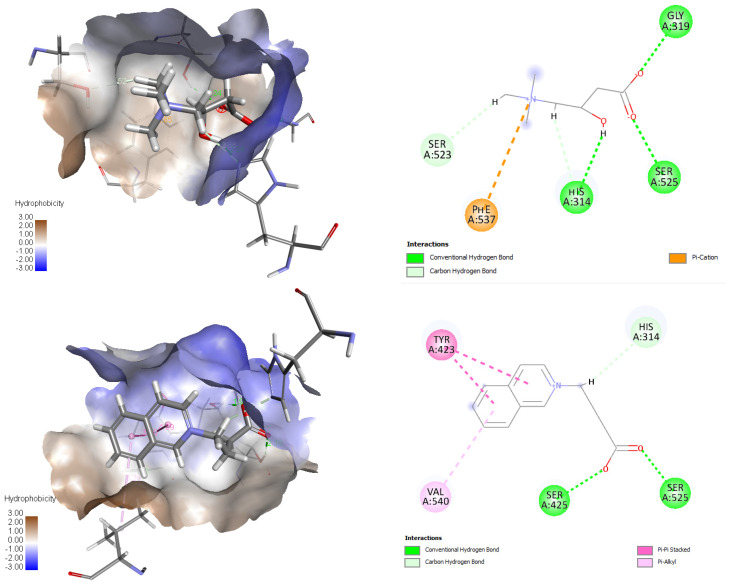
Three- and 2D visualizations of docking poses possessing the lowest binding energy (highest binding affinity) and specific interactions within the active center for the natural substrate L-carnitine (**upper**) and the most active compound **7** (**lower**) with CAT (*Mus musculus*, PDB identifier: 1NDI) [9]. The x, y, and z coordinates for the binding centers for CAT corresponded to His 314 (30.133, 34.746, 90.272). Results suggest that incorporating a heterocyclic ring as a substituent in the gamma-position of butanoic acid could be a prerequisite for reinforcing the hydrophobic and π–cation interactions within the enzyme’s hydrophobic pocket.

The results show that, aside from Mildronate and MeGBB, all betaines exhibited a stronger binding affinity than the natural substrate L-carnitine. Some aromatic compounds had values similar to their aliphatic analogs [e.g., pip-GBB (**2**) vs. pyr-GBB (**5**), pyrr-GBB (**1**) vs. mim-GBB (**4**)], suggesting that additional π–π interactions may not significantly affect activity. A trend of increasing affinity with larger substituents indicated enhanced hydrophobic interactions [mim-GBB (**4**) < pyr-GBB (**5**) < DMAP-GBB (**6**) < *i*-qui-GBB (**7**) ≈ qui-GBB (**8**)]. Compounds **7** and **8**, featuring the bulkiest substituents, isoquinoline and quinolone, respectively, showed the highest binding affinity with both enzymes. Unexpectedly, the ethyl ester salts yielded results similar to their corresponding betaines, contrary to established experimental data (see below). However, to support our hypothesis, we conducted further treatment and visualization of the automatically generated docking poses with the lowest binding energy (highest binding affinity) for the natural substrate L-carnitine (Figure 3, upper) and the most active compound **7** (Figure 3, lower) with CAT (*Mus musculus*, PDB identifier: 1NDI) [9]. These results demonstrated that the specific interactions within the active site, resulting from including a heterocyclic ring as a substituent in the gamma position of butanoic acid, enhance the hydrophobic interactions within the enzyme’s hydrophobic pocket. In summary, these preliminary theoretical calculations were considered a rational foundation for further experimental investigation into the potential of these compounds as competitive inhibitors of CAT.

### 2.2. Synthesis and Characterization

**Scheme 1:**
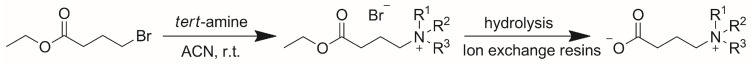
Synthesis of heterocyclic gamma-butyrobetaines. Substituents R^1^ and R^2^ are equivalent to R in Table 2 and R^3^- to R′.

The heterocyclic GBBs **1**–**8** were synthesized as per Scheme 1, following the procedure outlined by Tars and co-authors for preparing a wide range of acyclic aliphatic analogs of Meldonium [47]. The synthetic pathway involves a quaternization reaction of a chosen amine with ethyl 4-bromobutanoate, followed by hydrolysis. In the alkylation step, acetonitrile was utilized as the reaction medium. Being a polar aprotic solvent, it stabilized the transition state by redistributing charges that appeared during the reaction while simultaneously preventing the inactivation of the starting amine. In the case of aliphatic amines, *N*,*N*-dimethyl-4-aminopyridine and *N*-methylimidazole, this first step proceeded with excellent yields (>95%) under inert conditions at room temperature for 72 h. For isoquinoline and pyridine, the yields were notably lower (<50%) the latter being attributed to their reduced nucleophilicity; therefore, their alkylation was accomplished at an elevated temperature of 40–80 °C with an extended reaction time (5–7 days), resulting in yields of 65–74%. The salts obtained in this manner were further hydrolyzed using a strongly basic ion exchange resin (Amberlite IRA-410(Cl)), and after neutralization, the final products were obtained by treatment with a strongly acidic ion exchange resin (Amberlite IR120, hydrogen form, strongly acidic). Notably, the reaction with the quinoline, as well as with other heterocycles like oxazole and benzothiazole, resulted in a complex mixture with no measurable formation of the corresponding GBB-esters. As a result, we did not synthesize these compounds.

The so-outlined approach was not feasible for the conversion of 2-(4-ethoxy-4-oxobutyl)isoquinolin-2-yl bromide (**7**-ester) due to lower yields and the presence of additional impurities. Accordingly, 4-(isoquinolin-2-yl)butanoate (**7**) was synthesized by hydrolysis of **7**-ester in an acidic medium, subsequent neutralization with NaOH, conversion of the resulting NaBr and NaCl into their less soluble silver salts AgCl and AgBr, and the removal of the latter, as well as the additionally obtained NaNO_3_, by precipitation in water and suitable organic solvents.

All synthesized compounds are highly hygroscopic, making their characterization and preparation of solutions for biological assessment challenging. The latter was resolved by utilizing ^1^H-NMR spectroscopy with an internal standard of m-chlorobenzoic acid (m-CBA). Except for compounds **1**-ester, **3**, **3**-ester, **6**, and **7**, literature data are available for the chemical shifts in the ^1^H- and ^13^C-NMR spectra, which are consistent with our results [47,58,59,60,61,62]. In the proton spectra of all compounds, with the exceptions of 4-(isoquinolin-2-yl)butanoate (**7**) and 4-(pyridin-1-yl)butanoate (**5**), distinct peaks can be observed, including a multiplet around 2.0 ppm corresponding to the two hydrogen atoms of the methylene group in the beta-position relative to the nitrogen atom, a triplet around 2.20 ppm for the two hydrogen atoms from the methylene group in the alpha-position relative to the carboxyl group (or ester), and a triplet in the range of 3.0–3.5 ppm for aliphatic amines (or 3.5–4.0 ppm for aromatics), corresponding to the methylene group attached to the quaternary nitrogen atom. Two exceptions are compounds **5** and **7**, for which the latter signal is shifted beyond 4 ppm due to the nitrogen atom’s substantial partial positive charge (a group with a positive mesomeric effect is missing).

### 2.3. Biological Assessment

**Figure 4:**
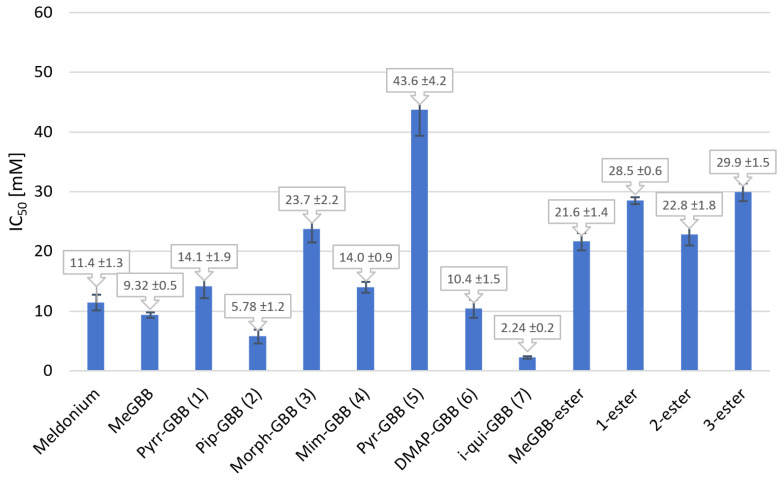
IC_50_ values of compounds **1–7**, four of the corresponding ethyl esters, and the positive controls Meldonium and MeGBB.

To assess the impact of the synthesized compounds on CAT activity and establish the structure-activity relationship, we initially determined the concentration needed to reduce the enzyme activity by 50% (IC_50_) under the same reaction conditions. We slightly modified a method proposed by Marquis and Fritz [63], which allows for the quantification of the consumed L-carnitine by observing its conversion to acetyl L-carnitine using a reaction with 5,5′-dithiobis-(2-nitrobenzoic acid) (DTNB or Ellman’s reagent) and CoA to produce 2-nitro-5-thiobenzoic acid (TNB). We opted to determine the IC_50_ at a substrate concentration that yields a [S]/*K*_m_ ratio of about 0.01 (c(L-carnitine) = 50.56 μM and *K*_m_ = 3.0 mM) to ensure that the IC_50_ is virtually independent of [S]. We also used a kinetic approach, measuring the reaction rate over a time interval Δt, during which the dependence is linear (approximately 10–15% substrate conversion). The readings were taken within the first minute after the reaction began, and the degree of inhibition was determined as described in Section 3.3.1. The IC_50_ values for each compound were obtained by analyzing the percentage inhibition at eight different inhibitor concentrations, with each measurement conducted in triplicate over three consecutive days. The average values of the IC_50_ obtained are outlined in Figure 4.

Based on the results depicted in Figure 4, it is evident that all the compounds under investigation possess inhibitory efficacy within the low millimolar range (IC_50_ = 2.24–43.6 mM), with certain compounds demonstrating superior activity compared to the designated positive standards, Meldonium and MeGBB (IC_50_ = 11.4 ± 1.32 and 9.32 ± 0.47 mM, respectively). The exploration of structure–activity relationships allows for the formulation of the following general conclusions: (1) The volume of the gamma-position substituent has a direct impact on the affinity of the compounds being tested since it correlates directly with the observed inhibition capacity. In the case of aromatic derivatives, the activity increases in the order pyr-GBB < mim-GBB < DMAP-GBB < *i*-qui-GBB, aligning with the increasing size of the substituents. Additionally, the pyrrolidine derivative inhibitor (pyrr-GBB) demonstrated a significantly higher IC_50_ value (14.1 ± 1.91 mM) compared to its six-membered piperidine derivative (pip-GBB, IC_50_ = 5.8 ± 1.15 mM). This is further supported by the inhibitory capacity of the compound with the bulkiest isoquinolinium fragment (*i*-qui-GBB), showing the highest inhibitory capacity (IC_50_ = 2.24 ± 0.21 mM). (2) The presence of a free carboxyl group is essential for enhanced activity. This relationship is evident when comparing the activities of GBB compounds with their respective esters, wherein the IC_50_ values are observed to be twice as high for the esters. (3) Increasing the polarity of the substituent in the gamma-position leads to a notable reduction in inhibitory ability. A comparison of the activity of *N*-methylmorpholine derivative (morph-GBB) with piperidine (pip-GBB) demonstrates this phenomenon. The presence of an oxygen atom in the morpholine ring increases the group’s polarity in comparison to piperidine, thereby constraining the inhibitor’s hydrophobic interactions within the enzyme’s hydrophobic pocket. (4) The presence of an aromatic substituent in the gamma-position does not serve as a crucial determinant of heightened activity. This is evidenced by the fact that inhibitors featuring an aromatic substituent demonstrate comparable or decreased affinity compared to their aliphatic counterparts. Based on the dependencies derived in this manner, one can conclude that the presence of a free carboxyl group and a bulky non-polar substituent in the gamma-position is a prerequisite for more effective interaction of the inhibitors with the active center of the enzyme.

While IC_50_ values serve as informative indicators for comparative studies and SAR analyses, they do not provide a comprehensive understanding of the specific interactions between inhibitors and enzymes. Therefore, we performed a kinetic study of the most active compound, 4-(isoquinolin-2-yl)butanoate (**7**), to acquire more profound insights, as outlined in Section 3.3.2. Substrate concentrations were selected in the range of 0.2 to five times the *K*_m_ value (*K*_m_ = 0.3 mM) to facilitate effective inhibition detection at elevated levels while ensuring sufficient substrate availability for the reaction at increased inhibitor concentrations. Notably, higher inhibitor concentrations (7.5 mM and 15 mM) resulted in deviations from the Michaelis–Menten model, so we worked at lower inhibitor concentrations (1.88 mM and 3.75 mM). Furthermore, at the lowest inhibitor concentration of 0.94 mM, the observed reaction rates were equivalent to those of blank samples, indicating a lack of inhibition; consequently, we excluded these values from subsequent analysis. The experiments enabled identification of the inhibition mechanism alongside calculations of *K*_m_, *V*_max_, and *K*_i_. We utilized SigmaPlot version 12.5 (Systat Software Inc. San Jose, CA, USA), which incorporates modules for regression analysis and various inhibition models. The numerical results are summarized in Table 3, while the corresponding Lineweaver –Burk and Michaelis–Menten kinetic plots are available in the Supplementary Materials. Our findings indicate that compound **7** functions as a competitive inhibitor of CAT (highest coefficient of determination (R^2^) and lowest AIC), exhibiting a *K*_i_ value of 3.5 mM, which is comparable to that of the drug Mildronate (*K*_i_ = 1.63 mM). This finding suggests the potential role of compound **7** as a metabolic regulator within the human body.

## 3. Materials and Methods

### 3.1. General

All chemicals and CAT (isolated from pigeon breast muscle, ammonium sulfate suspension, CAS Number: 9029-90-7) used in this study were purchased from Sigma-Aldrich (FOT, Sofia, Bulgaria). The organic solvents were of analytical grade and were used without further purification. Molecular docking was performed with the freely available online platform Mcule (Mcule Inc., Palo Alto, CA, USA; http://mcule.com). NMR spectra were recorded on a Bruker Avance III HD (Bruker BioSpin GmbH, Rheinstetten, Germany, 500 MHz and 126 MHz for ^1^H and ^13^C, respectively) and Bruker Avance II+ (600 MHz and 151 MHz for ^1^H and ^13^C, respectively) using D_2_O or CDCl_3_ as a solvents. The chemicals shifts (*δ*) are given in ppm and *J* values are reported in Hz. Column chromatography was performed on Horizon High Performance FLASH chromatography system (HPFC) with cartridges filled with Silica gel 60 [particle size—0.06–0.2 mm (70–230 mesh), MACHEREY-NAGEL, Düren, Germany]. Biological assessment was performed on ELISA Reader Biotek 800TS (Biotek Instruments, Inc, ELTA90, Sofia, Bulgaria). High-Resolution Mass Spectra (HRMS) were obtained on a Shimadzu LCMS-9050 (Shimadzu Handels GmbH., Korneuburg, Austria).

### 3.2. Synthesis

#### 3.2.1. Synthesis of Bromide Salts of Heterocyclic Gamma-Butyrobetaine Ethyl Esters

The corresponding *tert*-amine (1.1 equiv.) was added to a solution of ethyl-4-bromobutanoate in acetonitrile and the resulting mixture was stirred under inert atmosphere (nitrogen) for 72 h. at r.t. (22–23 °C), unless otherwise specified. At the end of the reaction, the unreacted amine and solvent were evaporated under reduced pressure, 250 mL of dry ethyl acetate was added to the residue, and the mixture was cooled to 0 °C for 20 min. The resulting crystalline bromide salts of gamma-butyrobetaine ethyl esters were isolated after filtration.

4-ethoxy-*N*-ethyl-*N,N*-dimethyl-4-oxobutan-1-aminium bromide (MeGBB-ester): *N,N*-dimethylethylamine (2.53 g, 34.6 mmol) reacted with ethyl-4-bromobutanoate (6.13 g, 31.4 mmol) to give white crystals of MeGBB-ester (8.34 g, 99% yield), m.p. 108–110 °C (lit. 80.9–99.0 °C, [47]). ^1^H-NMR (500 MHz, D_2_O): *δ* = 4.24–4.17 (2H, m, OC**H**_2_CH_3_), 3.42 (2H, q, ^3^*J* = 7.3 Hz, NC**H**_2_CH_3_), 3.38–3.28 (2H, m, 1-CH_2_), 3.08 (6H, s, NCH_3_), 2.53 (2H, t, ^3^*J* = 7.0 Hz, 3-CH_2_), 2.17–2.03 (2H, m, 2-CH_2_), 1.36 (3H, t, ^3^*J* = 7.2, NCH_2_C**H**_3_), 1.28 (3H, t, ^3^*J* = 7.2 Hz, OCH_2_C**H**_3_). ^13^C-NMR (126 MHz, D_2_O): *δ* = 174.63 (C, C=O), 62.10, 61.98, 59.66, 50.06, 50.03, 50.00, 30.23, 17.42, 13.29, 7.41. HRMS (ESI) *m*/*z* calculated for [M]^+^ C_10_H_22_NO_2_^+^: 188.16451; found: [M]^+^ 188.16397.

1-(4-ethoxy-4-oxobutyl)-1-methylpyrrolidin-1-ium bromide (pyrr-ester, **1**–ester): *N*-methylpyrrolidine (2.73 g, 32.1 mmol) reacted with ethyl-4-bromobutanoate (5.69 g, 29.2 mmol) to give white crystals of pyrr-ester (6.63 g, 81% yield), m.p. 117–119 °C (lit. 118–119 °C, [64]). ^1^H-NMR (600 MHz, D_2_O): *δ* = 4.15 (2H, q, ^3^*J* = 7.2 Hz, OC**H**_2_CH_3_), 3.58–3.43 (4H, m, 2′-CH_2_, 5′-CH_2_), 3.40–3.31 (2H, m, 1-CH_2_), 3.05 (3H, s, NCH3), 2.49 (2H, t, ^3^*J* = 7.1 Hz, 3-CH_2_), 2.24–2.15 (4H, m, 3′-CH_2_, 4′-CH_2_), 2.14–2.04 (2H, m, 2-CH_2_), 1.24 (3H, t, ^3^*J* = 7.2 Hz, OCH_2_C**H**_3_). ^13^C-NMR (151 MHz, D_2_O): *δ* = 174.77 (C=O), 64.49, 64.47, 64.45, 62.96, 62.05, 48.10, 30.46, 21.36, 18.72, 13.39. HRMS (ESI) *m*/*z* calculated for [M]^+^ C_11_H_22_NO_2_^+^: 200.16451; found: [M]^+^ 200.16409.

1-(4-ethoxy-4-oxobutyl)-1-methylpiperidin-1-ium bromide (pip-ester, **2**–ester): *N*-methylpiperidine (2.94 g, 29.7 mmol) reacted with ethyl-4-bromobutanoate (5.26 g, 27.0 mmol) to give white crystals of pip-ester (7.55 g, 95% yield), m.p. 111–112 °C (lit. 112 °C, [65]). ^1^H-NMR (600 MHz, D_2_O) *δ* = 4.16 (2H, q, ^3^*J* = 7.2 Hz, OC**H**_2_CH_3_), 3.38–3.30 (6H, m, 2′-CH_2_, 6′-CH_2_, 1-CH_2_), 3.03 (3H, s, NCH_3_), 2.48 (2H, t, ^3^*J* = 7.0 Hz, 3-CH_2_), 2.09–2.01 (2H, m, 2-CH_2_), 1.88–1.83 (4H, m, 3′-CH_2_, 5′-CH_2_), 1.68–1.59 (2H, m, 4′-CH_2_), 1.23 (3H, t, ^3^*J* = 7.2Hz, OCH_2_C**H**_3_). ^13^C-NMR (151 MHz, D_2_O): *δ* = 174.77 (C=O), 62.06, 61.29, 30.37, 20.57, 19.57, 16.82, 13.38. HRMS (ESI) *m*/*z* calculated for [M]^+^ C_12_H_24_NO_2_^+^: 214.18016; found: [M]^+^ 214.17950.

4-(4-ethoxy-4-oxobutyl)-4-methylmorpholin-4-ium bromide (morph-ester, **3**–ester): *N*-methylmorpholine (2.97 g, 29.4 mmol)) reacted with ethyl-4-bromobutanoate (5.21 g, 26.7 mmol) to give white crystals of morph-ester (4.82 g, 61% yield), m.p. 125–127 °C. ^1^H-NMR (600 MHz, D_2_O): *δ* = 4.20 (2H, q, ^3^*J* = 7.2 Hz, OC**H**_2_CH_3_), 4.10–4.05 (4H, m, 3′-CH_2_, 5′-CH_2_), 3.61–3.51 (6H, m, 2′-CH_2_, 6′-CH_2_, 1-CH_2_), 3.25 (3H, s, NCH_3_), 2.56 (2H, t, ^3^*J* = 7.0 Hz, 3-CH_2_), 2.19–2.10 (2H, m, 2-CH_2_), 1.30–1.26 (3H, t, ^3^*J* = 7.2 Hz, OCH_2_C**H**_3_). ^13^C-NMR (151 MHz, D_2_O): *δ* = 174.59 (C=O), 62.09, 60.46, 59.72, 30.19, 16.64, 13.38. HRMS (ESI) *m*/*z* calculated for [M]^+^ C_11_H_22_NO_3_^+^: 216.15942; found: [M]^+^ 216.15921.

#### 3.2.2. Synthesis of Heterocyclic Gamma-Butyrobetaines

The corresponding bromide salts of gamma-butyrobetaine ethyl esters obtained as described in Section 3.2.1. were dissolved in 3 mL dist. water and transferred to a column filled with strong base (Type II) anion exchange resin [Amberlite IRA-410(Cl), soaked in 10% (*wt*/*wt*) NaOH and washed with distilled water to pH = 7] in a ratio of 1/10 (*wt*/*wt*). After 1 h, the column was eluted with dist. water and the pH of the resulting solution was adjusted with strong acid cation exchange resin (Amberlite IR120 H) to neutral value (pH = 7). The resin was then filtered, and, after evaporation of the filtrate, the residue was azeotropically dried with abs. ethanol to give the corresponding gamma-butyrobetaine. This procedure was used for the synthesis of all compounds except for *i*-qui-GBB, which was synthesized using a modified procedure.

4-(ethyldimethylammonio)butanoate (MeGBB): Isolated as yellow-brown oil (4.95 g, 99% yield). ^1^H-NMR (500 MHz, D_2_O): *δ* = 3.41 (2H, q, ^3^*J* = 7.3 Hz, NC**H**_2_CH_3_), 3.34–3.25 (2H, m, 4-CH_2_), 3.07 (6H, s, NCH_3_), 2.30 (2H, t, ^3^*J* = 7.1 Hz, 2-CH_2_), 2.08–1.96 (2H, m, 3-CH_2_), 1.42–1.33 (3H, m, NCH_2_C**H**_3_). ^13^C-NMR (126 MHz, D_2_O): *δ* = 180.55 (C=O), 62.77, 59.65, 50.03, 50.00, 49.97, 33.33, 18.90, 7.43. HRMS (ESI) *m*/*z* calculated for [M+H]^+^ C_8_H_18_NO_2_^+^: 160.13321; found: [M+H]^+^ 160.13249.

4-(1-methyl-1H-imidazol-3-ium-3-yl)butanoate (mim-GBB, **4**): *N*-methylimidazole (2.67 g, 32.5 mmol) reacted with ethyl-4-bromobutanoate (5.76 g, 29.5 mmol) to white crystals of mim-ester (4.82 g, 61% yield), which, after hydrolysis, gave (4.57 g, 92%) yellow-brown oil of mim-GBB. ^1^H-NMR (500 MHz, D_2_O): *δ* = 7.38–7.21 (2H, m, 4′-H, 5′-H), 4.02 (2H, t, ^3^*J* = 7.1 Hz, 4-CH_2_), 3.72 (3H, s, NCH_3_), 2.06 (2H, t, ^3^*J* = 7.3 Hz, 2-CH_2_), 1.94 (2H, p, ^3^*J* = 7.1 Hz, 3-CH_2_). ^13^C-NMR (126 MHz, D_2_O): *δ* = 181.10 (C, C=O), 130.78, 123.46, 122.08, 48.94, 35.59, 33.73, 26.15. HRMS (ESI) *m*/*z* calculated for [M+H]^+^ C_8_H_13_N_2_O_2_^+^: 169.09715; found: [M+H]^+^ 169.09683.

4-(pyridin-1-ium-1-yl)butanoate (pyr-GBB, **5**):. Pyridine (2.62 g, 33.1 mmol) reacted with ethyl-4-bromobutanoate (5.87 g, 30.1 mmol) at 40°C for 5 days to white crystals of pyr-ester (3.80 g, 65% yield), which, after hydrolysis, gave pyr-GBB as yellow-brown crystals (2.5 g, 50% yield), m.p. 63–65 °C (197–198 °C [58]; lit. 98–99 °C, [66]). ^1^H-NMR (500 MHz, D_2_O): *δ* = 8.87 (2H, d, ^3^*J* = 5.5 Hz, 2′-H, 6′-H), 8.59–8.52 (1H, m, 4′-H), 8.08 (1H, t, ^3^*J* = 7.1 Hz, 3′-H, 5′-H), 4.66–4.61 (2H, m, 4-CH_2_), 2.29–2.25 (4H, m, 3-CH_2_, 2-CH_2_). ^13^C-NMR (126 MHz, D_2_O): *δ* = 180.66 (C=O), 145.62, 144.30, 128.20, 61.39, 33.56, 27.34. HRMS (ESI) *m*/*z* calculated for [M+H]^+^ C_9_H_12_NO_2_^+^: 166.08626; found: [M+H]^+^ 166.08554.

4-(4-(dimethylamino)pyridin-1-ium-1-yl)butanoate (DMAP-GBB, **6**): *N,N*-dimethyl-4-aminopyridine (9.62 g, 30.3 mmol) reacted with ethyl-4-bromobutanoate (5.38 g, 27.6 mmol) to white crystals of DMAP-ester (6.48 g, 99% yield), which, after hydrolysis, gave DMAP-GBB as yellow-brown oil (4.88 g, 98% yield). ^1^H-NMR (500 MHz, D_2_O): *δ* = 7.85 (2H, d, ^3^*J* = 6.8 Hz, 2′-H, 6′-H), 6.67 (2H, d, ^3^*J* = 6.8 Hz, 3′-H, 5′-H), 4.03 (2H, t, ^3^*J* = 7.1 Hz, 4-CH_2_), 3.05 (s, 6H; NCH_3_), 2.15 (2H, t, ^3^*J* = 7.3 Hz, 2-CH_2_), 2.01 (2H, p, ^3^*J* = 7.1 Hz, 3-CH_2_). ^13^C-NMR (126 MHz, D_2_O): *δ* = 181.17 (C=O), 141.10, 107.41, 57.06, 39.24, 33.73, 26.90. HRMS (ESI) *m*/*z* calculated for [M+H]^+^ C_11_H_17_N_2_O_2_^+^: 209.12845; found: [M+H]^+^ 209.12827.

4-(isoquinolin-2-ium-2-yl)butanoate (*i*-qui-GBB, **7**): Isoquinoline (1 g, 7.74 mmol) reacted with ethyl-4-bromobutanoate (3.02 g, 15.48 mmol) at 80 °C for 7 days to give a complex oily mixture. The bromide salt of *i*-qui-GBB-ester was purified via flash chromatography (using MeOH as mobile phase), dissolved and stirred in 20 mL 2% HCl for 24 h. The resulting mixture was then neutralized with 10% NaOH, and 50% aqueous solution of AgNO_3_ was added dropwise until no observable formation of AgCl or AgBr occurred. The inorganic salts were filtered, the solvent was evaporated under reduced pressure, and abs. ethanol was added to the dry oily residue. The formed crystals of NaNO_3_ were then filtered and the solvent was evaporated under reduced pressure to give *i*-qui-GBB (1.12 g, 67% yield), m.p. 149–152 °C. ^1^H-NMR (600 MHz, CDCl_3_): *δ* = 12.03 (1H, s, 1′-CH), 10.86 (1H, m, 3′-CH), 10.74–10.68 (2H, m, 4′-CH, 8′-CH), 10.55–10.50 (2H, m, 5′-CH, 6′-CH), 10.38–10.33 (1H, m, 7′-CH), 7.12 (2H, t, ^3^*J* = 7.1 Hz, 4-CH_2_), 4.74–4.68 (4H, m, 2-CH_2_, 3-CH_2_). ^13^C-NMR (151 MHz, CDCl_3_): *δ* = 183.13 (C=O), 151.57, 139.77, 139.44, 136.41, 133.74, 132.43, 129.93, 129.54, 128.86, 63.52, 36.11, 29.87. HRMS (ESI) *m*/*z* calculated for [M+H]^+^ C_13_H_14_NO_2_^+^: 216.10191; found: [M+H]^+^ 216.10170.

4-(1-methylpyrrolidin-1-ium-1-yl)butanoate (Pyrr-GBB, **1**): *N*-methylpyrrolidine (2.73 g, 32.1 mmol) reacted with ethyl-4-bromobutanoate (5.69 g, 29.2 mmol) to white crystals of pyrr-ester (6.63 g, 81% yield), which, after hydrolysis, gave yellow-brown oil of pyrr-GBB (4.05 g, 81% yield). ^1^H-NMR (600 MHz, D_2_O): *δ* = 3.51–3.38 (4H, m, 2′-CH_2_, 5′- CH_2_), 3.29–3.23 (2H, m, 4-CH_2_), 2.99 (3H, s, NCH_3_), 2.20 (2H, t, ^3^*J* = 7.2 Hz, 2-CH_2_), 2.18–2.10 (4H, m, 3′-CH2, 4′-CH2), 2.02–1.94 (2H, m, 3-CH_2_). ^13^C-NMR (151 MHz, D_2_O): *δ* = 180.83 (C=O), 64.37, 64.35, 64.33, 63.60, 63.58, 48.04, 48.01, 47.99, 33.74, 21.30, 20.26. HRMS (ESI) *m*/*z* calculated for [M+H]^+^ C_9_H_18_NO_2_^+^: 172.13321; found: [M+H]^+^ 172.13246.

4-(1-methylpiperidin-1-ium-1-yl)butanoate (pip-GBB, **2**): *N*-methylpiperidine (2.94 g, 29.7 mmol) reacted with ethyl-4-bromobutanoate (5.26 g, 27.0 mmol) to white crystals of pip-ester (7.55 g, 95% yield), which, after hydrolysis, gave yellow-brown oil of pip-GBB (4.45 g, 89% yield). ^1^H-NMR (600 MHz, D_2_O): *δ* = 3.35–3.23 (6H, m, 2′-CH2, 6′-CH2, 4-CH_2_), 2.99 (3H, s, NCH_3_), 2.21 (2H, t, ^3^*J* = 7.1 Hz, 2-CH_2_), 1.98–1.90 (2H, m, 3-CH_2_), 1.82 (4H, p, ^3^*J* = 5.7 Hz, 3′-CH2, 5′-CH_2_), 1.66–1.55 (2H, m, 4′-CH_2_). ^13^C-NMR (151 MHz, D_2_O): *δ* = 180.87 (C=O), 62.54, 61.21, 47.85, 33.65, 20.59, 19.53, 18.33. HRMS (ESI) *m*/*z* calculated for [M+H]^+^ C_10_H_20_NO_2_^+^: 186.14886; found: [M+H]^+^ 186.14811.

4-(4-methylmorpholino-4-ium)butanoate (morph-GBB, **3**): *N*-methylmorpholine (2.97 g, 29.4 mmol) reacted with ethyl-4-bromobutanoate (5.21 g, 26.7 mmol) to white crystals of morph-ester (4.82 g, 61% yield), which, after hydrolysis, gave yellow-brown oil of morph-GBB (2.65 g, 53% yield). ^1^H-NMR (600 MHz, D_2_O): *δ* = 4.00 (4H, t, ^3^*J* = 4.7 Hz, 3′-CH_2_, 5′-CH_2_), 3.52–3.39 (6H, m, 2′- CH_2_, 6′- CH_2_, 4-CH_2_), 3.16 (3H, s, NCH_3_), 2.23 (2H, t, ^3^*J* = 7.1 Hz, 2-CH_2_), 2.03–1.94 (2H, m, 3-CH_2_). ^13^C-NMR (151 MHz, D_2_O): *δ* = 180.63 (C=O), 64.39, 60.43, 59.66, 46.94, 33.45, 18.16. HRMS (ESI) *m*/*z* calculated for [M+H]^+^ C_9_H_18_NO_3_^+^: 188.12812; found: [M+H]^+^ 188.12794.

### 3.3. In Vitro Studies

Carnitine acetyltransferase isolated from pigeon breast muscle, ammonium sulfate suspension (CAS Number: 9029-90-7), with activity 71 U/mg protein (1U converts 1 μmol L-carnitine and 1 μmol AcCoA in Acetylcarnitine and free CoA in 1 min) was diluted with phosphate buffer (0.5 M, pH = 7.6) to obtain stock solution with concentration 24 U/mL. Ellman reagent was freshly prepared just before use by dissolving 25 mg DTNB in 5 mL 1 mM solution of Na_2_EDTA in phosphate buffer (0.5 M, pH = 7.6), the final concentration of DTNB in this solution being 12.8 mM. AcCoA, L-carnitine, and each of the tested compounds were dissolved in distilled water in order to obtain desired stock solutions with concentration c (AcCoA) = 348.0 μM and c (L-carnitine) = 303.4 μM (for IC_50_ determination). Tris-buffer solution was prepared by dissolving Tris. HCl in distilled water and pH was adjusted with NaOH to pH = 7.6, the final concentration of the solution being 1 M. The final volume of the reaction mixture was 300 μL, which included 50 μL of each stock solution of CAT, AcCoA, L-carnitine, DTNB, Tris-buffer, and test compound. In control probes, 50 μL distilled water was added instead of test compound. A mixture of CAT, AcCoA, DTNB, Tris-buffer, and test compounds were incubated for 5 min at 37 °C. The reaction was initiated by adding L-carnitine, and the reaction rate was monitored by measuring the absorbance at 405 nm.

#### 3.3.1. IC_50_ Determination

Inhibition activity was assessed by calculating the reaction rate during the initial minute, within the linear range of A/t, utilizing the following formula:

%Inh = 100 × ((ΔA/Δt)control − (ΔA/Δt) Inh)/((ΔA/Δt) control)

To obtain preliminary data for the IC_50_ values, we initially screened each compound at a final concentration of 5 mM. Then, we prepared eight concentrations of each test compound, ranging from 20% to 80% of the IC_50_, for further testing. The half-maximal inhibitory concentration (IC_50_) was determined by plotting the response (A) against the concentration (c) of the inhibitor. All analyses were performed in triplicate over three consecutive days, and the corresponding IC_50_ values were averaged.

#### 3.3.2. Enzyme Kinetics

The mechanism of inhibition for the most active compound was elucidated using five concentrations of the substrate L-carnitine (ranging from 20% to 80% of *K*_m_), and two concentrations of the inhibitor. For each substrate concentration, three separate reactions were performed: two with different concentrations of the inhibitor (1.88 mM and 3.75 mM) and one without it, the latter serving as a control. The absorbance at 405 nm was measured every 6 s between the 30th second and the 3rd minute for all probes (in the linear range). The initial velocity (*v*_0_) was determined as the intercept of the velocity (*v*) versus time (*t*) graph. All experiments were conducted in triplicate over three consecutive days. Nonlinear regression analysis was performed using SigmaPlot, version 12.5 (Systat Software Inc., San Jose, CA, USA). Graphical dependencies are provided in the Supplementary Materials (Figures S1 and S2).

### 3.4. Molecular Docking

Binding energies (kcal/mol) for the compounds studied herein were automatically computed using the 1-Click docking tool available on the Mcule online platform [https://mcule.com (accessed on 27 January 2025, Mcule Inc., Palo Alto, CA, USA)] [56]. This online platform incorporates an embedded version of AutoDock Vina for docking operations [67] and utilizes AutoDock tools to prepare both ligand and target for the docking process [68]. Detailed instructions on using the 1-Click docking tool, including a tutorial, can be found at https://mcule.com/apps/1-click-docking/ (accessed on 27 January 2025). The target proteins were selected from the Mcule database—CAT isolated from mouse (*Mus musculus*, PDB identifier: 1NDI) [9] and CPT2 isolated from rat (*Rattus norvegicus*, PDB identifier: 2FW3) [57]. The x, y, and z coordinates for the binding center of CAT corresponded to His 314 (30.133, 34.746, 90.272), while these for the CPT2 were default values (18.0041, 6.6764, 32.0234). We considered protein molecules as rigid structures without water molecules, while the ligands were flexible and optimized by the 1-Click docking algorithm. Twelve poses were generated for each compound, and those with the lowest binding energy (indicating the highest binding affinity) were selected and listed in Table 1. The poses of the natural substrate L-carnitine and the most active compound **7** with CAT (*Mus musculus*, PDB identifier: 1NDI) [9] were further evaluated and visualized using BIOVIA Discovery Studio Visualizer v24.1.0.23298 [69]. The poses demonstrating the highest binding affinity and the specific interactions within the active center are shown in Figure 3.

## 4. Conclusions

Utilizing a combined approach of rational design, molecular docking, and in vitro studies, we showed the significant potential to serve as metabolic modulators for a series of heterocyclic analogs of gamma-butyrobetaine. These compounds exhibit the capability to regulate FAO through the inhibition of CAT, and perhaps other enzymes within the CT family. Comparative analysis has revealed that all compounds tested demonstrate inhibitory potential within the low millimolar region (IC_50_ = 2.24–43.6 mM), with some exhibiting greater activity than established standards such as Meldonium (marketed as Mildronate^®^, an approved drug in several European countries for the treatment of heart ischemia, with IC_50_ = 11.39 ± 1.32 mM) and MeGBB (a drug candidate in phase III of clinical trials, with IC_50_ = 9.32 ± 0.47 mM). The determined IC_50_ values have facilitated additional insights into the structure–activity relationship: (1) the aromatic nature of the substituent in the gamma-position does not exert a significant influence on activity; (2) esterification of the carboxyl group, alongside an increase in the polarity of the substituent in the gamma position, markedly diminishes inhibitory ability; (3) the augmentation in the volume of substituents in the gamma-position correlates directly with the observed inhibition capacity. Further kinetic studies have identified the most active compound as a reversible competitive inhibitor of CAT with a *K*_i_ value of 3.5 mM, a level of inhibition comparable to the established drug Mildronate (*K*_i_ = 1.63 ± 0.52 mM).

In conclusion, this study suggests the potential of the heterocyclic GBB analogs as agents for regulating metabolic processes in the human body and for treating ischemic disease, diabetes, and certain cancer types. Current experiments are being conducted to evaluate the toxicity and in vivo effects of selected compounds, and the results will be presented in due course.

## Figures and Tables

**Table 1:** Fragments of the amino acid (AA) sequences of human, mouse, pigeon, and rat CT, showing those AAs directly involved in the binding of L-carnitine. The residues in bold/underlined have a key role in binding L-carnitine to the enzyme’s active site.

.	AA Sequence	AA Position	AA Sequence	AA Position	AA Sequence	AA Position
Human (CAT) [8,12]	T**Y**ESASL**R**MFHLGR**T**D	430–445	GEAFD**R**HLLGL	492–502	DCVMF**F**GP**V**VP	540–550
Mouse (CAT) [9]	T**Y**ESASL**R**MFHLGR**T**D	451–466	GEAFD**R**HLLGL	513–523	DCVMF**F**GP**V**VP	561–571
Mouse (CAT) [56]	T**Y**ESASL**R**MFHLGR**T**D	422–437	GEAFD**R**HLLGL	484–494	DCVMF**F**GP**V**VP	532–542
Pigeon (CAT) [55]	T**Y**ESASL**R**MFRLGR**T**D	453–468	GNAID**R**HLLGL	514–524	DCVMC**F**GP**V**VP	562–572
Human (COT) [8]	C**Y**ETAMT**R**HFYHGR**T**E	438–453	GKGFD**R**HLLGL	500–510	GYLRVQGV**V**VP	548–558
Human (CPT1) [8]	T**Y**EASMT**R**LFREGR**T**E	588–603	GSGID**R**HLFCL	650–660	SSGGG**F**GP**V**AP	706–716
Human (CPT2) [13]	T**Y**ESCSTAAFKHGR**T**E	485–500	GQGFD**R**HLFAL	549–559	VNLGG**F**AP**V**VS	598–608
Rat (CPT2) [56]	T**Y**ESCSTAAFKHGR**T**E	454–469	GQGFD**R**HLFAL	518–528	VSLGG**F**AP**V**VP	566–576

**Table 2:** Numbering, abbreviation, substitution pattern, and theoretically calculated affinity of the studied compounds (**1**–**8**): R, heterocyclic system; R′, substituent attached to the quaternary nitrogen atom outside the ring; X, substituent attached to the carbon atom of the acyl group; A^−^, anion. Lower absolute value denotes a higher binding affinity of the inhibitor to the active site of CAT isolated from mouse (*Mus musculus*, PDB identifier: 1NDI) [9] and CPT2 isolated from rat (*Rattus norvegicus*, PDB identifier: 2FW3) [57].

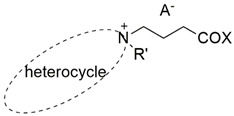							
		Substituents and Anion				Binding Energy (kcal/mol)	
Compd. No	Abbr.	R	R′	X	A^−^	CAT	CPT2
	L-carnitine					−4.5	−4.5
	Meldonium					−4.5	−4.4
	MeGBB		-CH_3_	O^−^	-	−4.6	−4.7
	MeGBB-ester		-CH_3_	-OEt	Br^−^	−4.8	−5.0
**1**	Pyrr-GBB	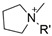	-CH_3_	O^−^	-	−5.3	−5.4
	**1**-ester		-CH_3_	-OEt	Br^−^	−5.3	−5.4
**2**	Pip-GBB	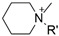	-CH_3_	O^−^	-	−5.4	−5.6
	**2**-ester		-CH_3_	-OEt	Br^−^	−5.8	−5.5
**3**	Morph-GBB	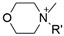	-CH_3_	O^−^	-	−5.1	−5.2
	**3**-ester		-CH_3_	-OEt	Br^−^	−5.4	−5.6
**4**	Mim-GBB	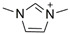	-	O^−^	-	−5.1	−5.3
	**4**-ester		-	-OEt	Br^−^	−5.2	−5.1
**5**	Pyr-GBB	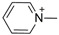	-	O^−^	-	−5.7	−5.5
	**5**-ester		-	-OEt	Br^−^	−5.7	−6.1
**6**	DMAP-GBB	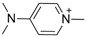	-	O^−^	-	−6.0	−6.3
	**6**-ester		-	-OEt	Br^−^	−5.9	−6.4
**7**	*i*-qui-GBB	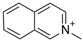	-	O^−^	-	−7.2	−7.2
	**7**-ester		-	-OEt	Br^−^	−7.2	−7.3
**8**	Qui-GBB	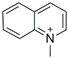	-	O^−^	-	−7.1	−6.7
	**8**-ester		-	-OEt	Br^−^	−7.2	−6.8

**Table 3:** Coefficient of determination (R^2^), Akaike Information Criterion (AIC), and standard deviation of the residuals (S_y,x_) for the different types of enzyme inhibition based on kinetic measurement of CAT activity in the presence of compound **7**.

Type of Inhibition	R^2^	AIC	S_y.x_
Competitive	0.99382	−1710.565	5.215 × 10^−9^
Noncompetitive	0.97754	−1652.521	9.938 × 10^−9^
Noncompetitive (partial)	0.97754	−1649.983	1.006 × 10^−8^
Uncompetitive	0.95923	−1625.683	1.339 × 10^−8^
Uncompetitive (partial)	0.95922	−1623.139	1.355 × 10^−8^

## Data Availability

The original contributions presented in this study are included in the Supplementary Material. Further inquiries can be directed to the corresponding author.

## Supplementary Materials

The following supporting information can be downloaded at: https://www.mdpi.com/article/10.3390/molecules30030735/s1. Michaelis–Menten and Lineweaver–Burk plots for different types of inhibition; ^1^H- and ^13^C-NMR spectra of all compounds described in the script.
