# Supplementary material for: Rational Design, Synthesis, and In Vitro Activity of Heterocyclic Gamma-Butyrobetaines as Potential Carnitine Acetyltransferase Inhibitors
Source: Molecules. 2025 Feb 6;30(3):735. doi: 10.3390/molecules30030735 (PMC11820905; doi:10.3390/molecules30030735)
Supplement: Supplementary file 1 [file molecules-30-00735-s001.zip › Supplementary Materials_v4.pdf]

Supplementary Material

Rational design, synthesis and in vitro activity of heterocyclic gamma-butyrobetaines as potential Carnitine Acetyltransferase inhibitors

Savina Stoyanova, Milen G. Bogdanov

Content:

Figure S1. The dependence of the reaction rate on time. Speed decreases linearly from 36 seconds to the end of the measurement - 180 seconds.

Figure S2. Graphs representing the dependence of the initial velocity (v_0) on the concentration of the substrate [S], according to the model of Michaelis-Menten – left, and Lineweaver-Burk – right, for reversible a) competitive, b) noncompetitive and c) uncompetitive inhibition.

Figure S3. ^{1}H-NMR Spectrum of 4-ethoxy-N-ethyl-N,N-dimethyl-4-oxobutan-1-aminium bromide (MeGBB-ester) in D_2O-d_2

Figure S3a. ^{1}H-NMR Spectrum of 4-ethoxy-N-ethyl-N,N-dimethyl-4-oxobutan-1-aminium bromide (MeGBB-ester) in D_2O-d_2.

Figure S4. ^{13}C-NMR Spectrum of 4-ethoxy-N-ethyl-N,N-dimethyl-4-oxobutan-1-aminium bromide (MeGBB-ester) in D_2O-d_2.

Figure S5. ^{1}H-NMR Spectrum of 4-(ethyldimethylammonio)butanoate (MeGBB) in D_2O-d_2.

Figure S5a. ^{1}H-NMR Spectrum 4-(ethyldimethylammonio)butanoate (MeGBB) in D_2O-d_2.

Figure S6. ^{13}C-NMR Spectrum of 4-(ethyldimethylammonio)butanoate (MeGBB) in D_2O-d_2.

Figure S7. ^{1}H-NMR Spectrum of 1-(4-ethoxy-4-oxobutyl)-1-methylpyrrolidin-1-ium (Pyrr-ester) in D_2O-d_2.

Figure S7a. ^{1}H-NMR Spectrum of 1-(4-ethoxy-4-oxobutyl)-1-methylpyrrolidin-1-ium (Pyrr-ester) in D_2O-d_2.

Figure S8. ^{13}C-NMR Spectrum of 1-(4-ethoxy-4-oxobutyl)-1-methylpyrrolidin-1-ium (Pyrr-ester) in D_2O-d_2.

Figure S9. ^{1}H-NMR Spectrum of 4-(1-methylpyrrolidin-1-ium-1-yl)butanoate (Pyrr-GBB) in D_2O-d_2.

Figure S9a. ^{1}H-NMR Spectrum of 4-(1-methylpyrrolidin-1-ium-1-yl)butanoate (Pyrr-GBB) in D_2O-d_2.

Figure S10. ^{13}C-NMR Spectrum of 4-(1-methylpyrrolidin-1-ium-1-yl)butanoate (Pyrr-GBB) in D_2O-d_2

Figure S11. ^{1}H-NMR Spectrum of 1-(4-ethoxy-4-oxobutyl)-1-methylpiperidin-1-ium bromide (Pip-ester) in D_2O-d_2.

Figure S11a. ^{1}H-NMR Spectrum of 1-(4-ethoxy-4-oxobutyl)-1-methylpiperidin-1-ium bromide (Pip-ester) in D_2O-d_2.

Figure S12. ^{13}C-NMR Spectrum of 1-(4-ethoxy-4-oxobutyl)-1-methylpiperidin-1-ium bromide (Pip-ester) in D_2O-d_2.

Figure S13. ^{1}H-NMR Spectrum of 4-(1-methylpiperidin-1-ium-1-yl)butanoate (Pip-GBB) in D_2O-d_2.

Figure S13a. ^{1}H-NMR Spectrum of 4-(1-methylpiperidin-1-ium-1-yl)butanoate (Pip-GBB) in D_2O-d_2.

Figure S14. ^{13}C-NMR Spectrum of 4-(1-methylpiperidin-1-ium-1-yl)butanoate (Pip-GBB) in D_2O-d_2.

Figure S15. ^{1}H-NMR Spectrum of 4-(4-ethoxy-4-oxobutyl)-4-methylmorpholin-4-ium (Morph-ester) in D_2O-d_2.

Figure S15a. ^{1}H-NMR Spectrum of 4-(4-ethoxy-4-oxobutyl)-4-methylmorpholin-4-ium (Morph-ester) in D_2O-d_2.

Figure S16. ^{13}C-NMR Spectrum of 4-(4-ethoxy-4-oxobutyl)-4-methylmorpholin-4-ium (Morph-ester) in D_2O-d_2.

Figure S17. ^{1}H-NMR Spectrum of 4-(4-methylmorpholino-4-ium)butanoate (Morph-GBB) in D_2O-d_2.

Figure S17a. ^{1}H-NMR Spectrum of 4-(4-methylmorpholino-4-ium)butanoate (Morph-GBB) in D_2O-d_2.

Figure S18. ^{13}C-NMR Spectrum of 4-(4-methylmorpholino-4-ium)butanoate (Morph-GBB) in D_2O-d_2.

Figure S19. ^{1}H-NMR Spectrum of 4-(1-methyl-1H-imidazol-3-ium-3-yl)butanoate (Mim-GBB) in D_2O-d_2.

Figure S19a. ^{1}H-NMR Spectrum of 4-(1-methyl-1H-imidazol-3-ium-3-yl)butanoate (Mim-GBB) in D_2O-d_2.

Figure S20. ^{13}C-NMR Spectrum of 4-(1-methyl-1H-imidazol-3-ium-3-yl)butanoate (Mim-GBB) in D_2O-d_2.

Figure S21. ^{1}H-NMR Spectrum of 4-(pyridin-1-ium-1-yl)butanoate (Pyr-GBB) in D_2O-d_2.

Figure S21a. ^{1}H-NMR Spectrum 4-(pyridin-1-ium-1-yl)butanoate (Pyr-GBB) in D_2O-d_2.

Figure S22. ^{13}C-NMR Spectrum of 4-(pyridin-1-ium-1-yl)butanoate (Pyr-GBB) in D_2O-d_2.

Figure S23. ^{1}H-NMR Spectrum of 4-(4-(dimethylamino)pyridin-1-ium-1-yl)butanoate (DMAP-GBB) in D_2O-d_2.

Figure S23a. ^{1}H-NMR Spectrum of 4-(4-(dimethylamino)pyridin-1-ium-1-yl)butanoate (DMAP-GBB) in D_2O-d_2.

Figure S24. ^{13}C-NMR Spectrum of 4-(4-(dimethylamino)pyridin-1-ium-1-yl)butanoate (DMAP-GBB) in D_2O-d_2.

Figure S25. ^{1}H-NMR Spectrum of 4-(isoquinolin-2-ium-2-yl)butanoate (*i*-qui-GBB) in $CDCl_3$-d_1.

Figure S25a. ^{1}H-NMR Spectrum of 4-(isoquinolin-2-ium-2-yl)butanoate (*i*-qui-GBB) in $CDCl_3$-d_1.

Figure S26. ^{13}C-NMR Spectrum of 4-(isoquinolin-2-ium-2-yl)butanoate (*i*-qui-GBB) in $CDCl_3$-d_1.

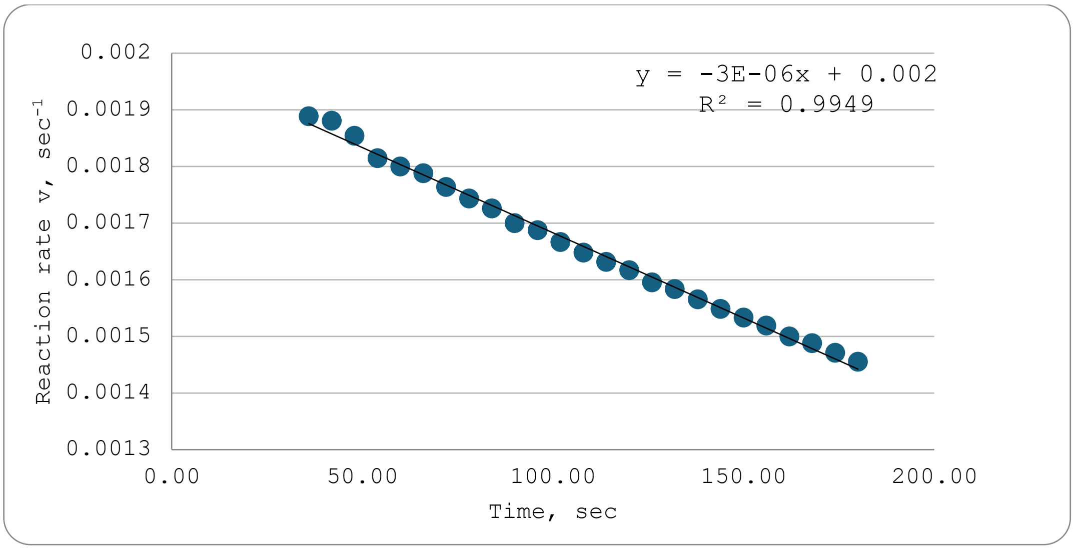

Figure S1. The dependence of the reaction rate on time (c(Inh) = 0, c(L-carnitine) = 359.53mM). Speed decreases linearly from 36 seconds to the end of the measurement - 180 seconds.

Figure S2 (next three pages). Graphs representing the dependence of the initial velocity (v_0) on the concentration of the substrate [S], according to the model of Michaelis-Menten – left, and Lineweaver-Burk – right, for reversible a) competitive, b) noncompetitive and c) uncompetitive inhibition. The concentration of the inhibitor - compound 7 are 0 mM, 1.875 mM and 3.75 mM, concentration of L-carnitine are 14 mM, 36 mM, 72 mM, 140 mM and 360 mM.

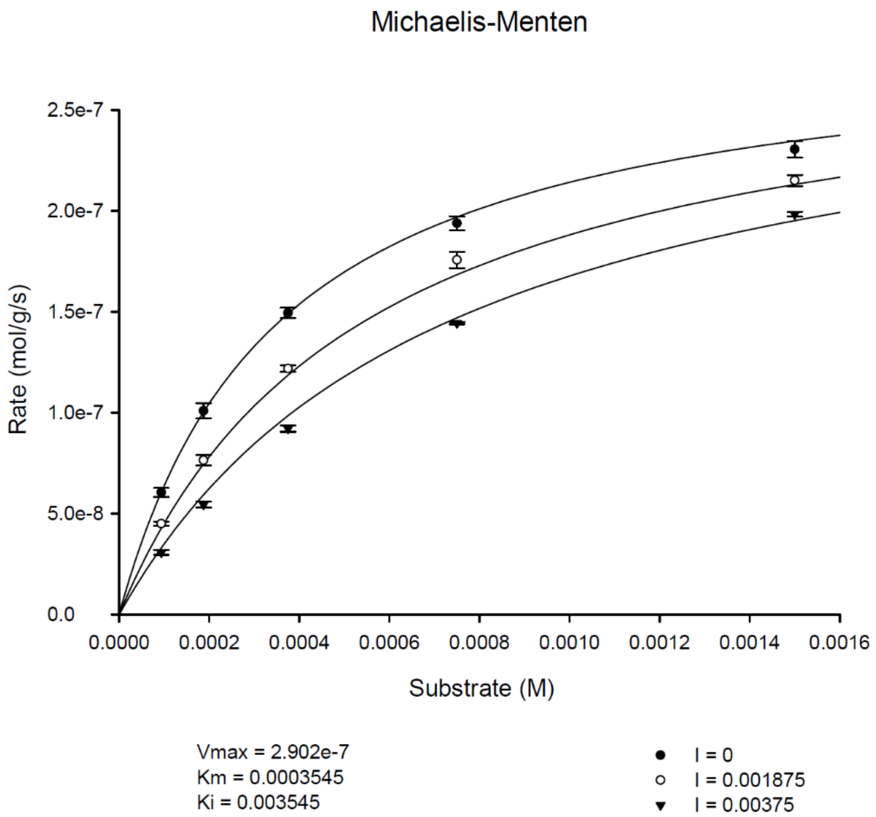
Michaelis-Menten
2.5e-7
2.0e-7
1.5e-7
1.0e-7
5.0e-8
0.0
Rate (mol/g/s)
0.0000
0.0002
0.0004
0.0006
0.0008
0.0010
0.0012
0.0014
0.0016
Substrate (M)
Vmax = 2.902e-7
Km = 0.0003545
Ki = 0.003545
I = 0
I = 0.001875
I = 0.00375

Fig. S2 a)

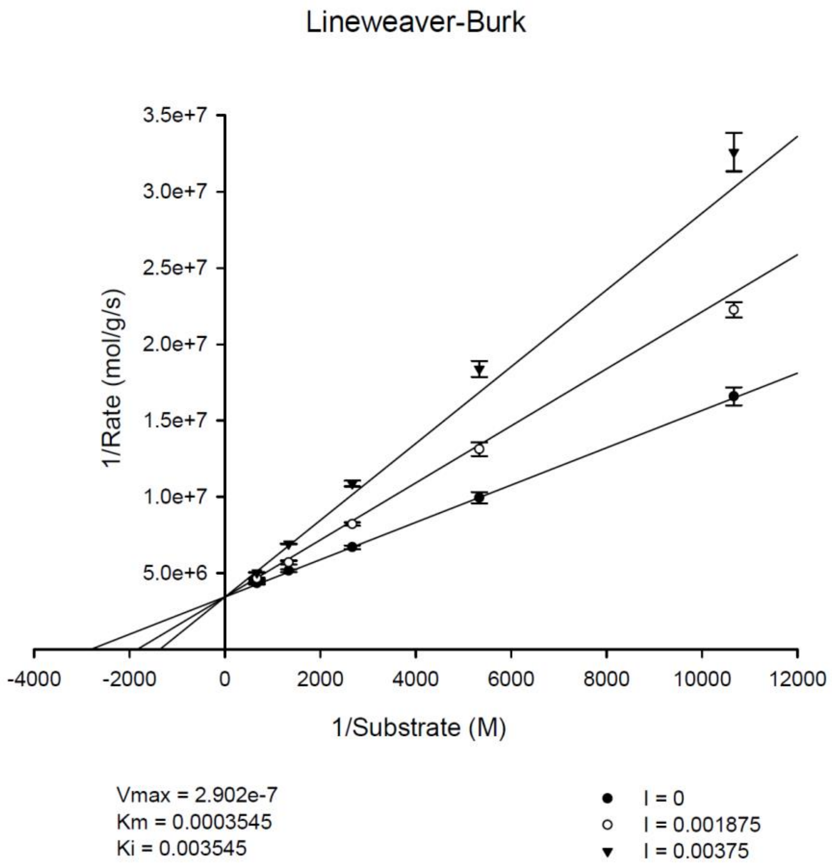
Lineweaver-Burk
3.5e+7
3.0e+7
2.5e+7
2.0e+7
1.5e+7
1.0e+7
5.0e+6
1/Rate (mol/g/s)
-4000
-2000
0
2000
4000
6000
8000
10000
12000
1/Substrate (M)
Vmax = 2.902e-7
Km = 0.0003545
Ki = 0.003545
I = 0
I = 0.001875
I = 0.00375

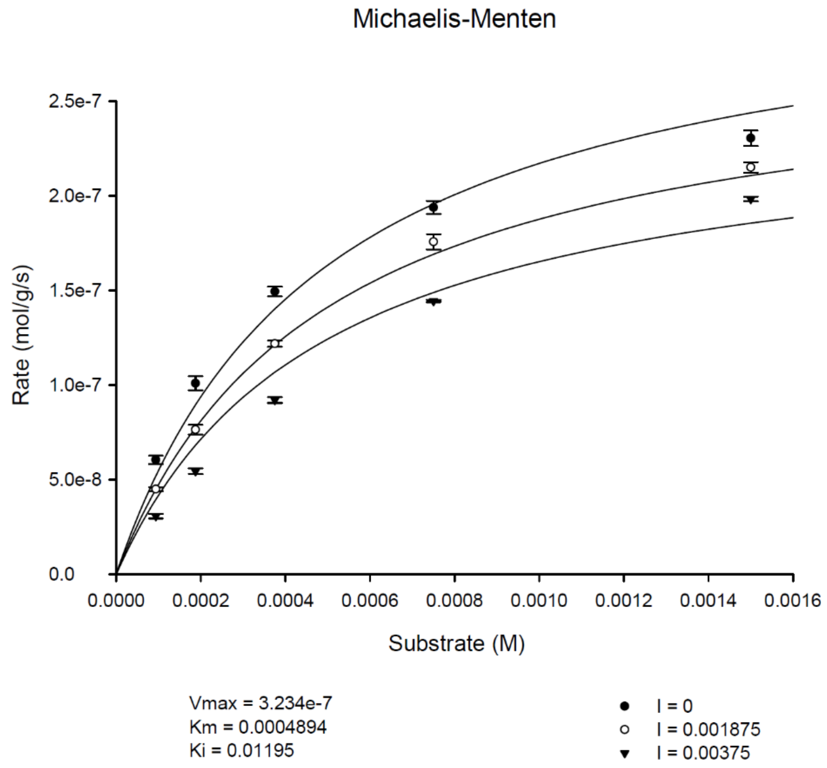
Michaelis-Menten
Rate (mol/g/s)
2.5e-7
2.0e-7
1.5e-7
1.0e-7
5.0e-8
0.0
0.0000
0.0002
0.0004
0.0006
0.0008
0.0010
0.0012
0.0014
0.0016
Substrate (M)
Vmax = 3.234e-7
Km = 0.0004894
Ki = 0.01195
I = 0
I = 0.001875
I = 0.00375

Fig. S2 b)

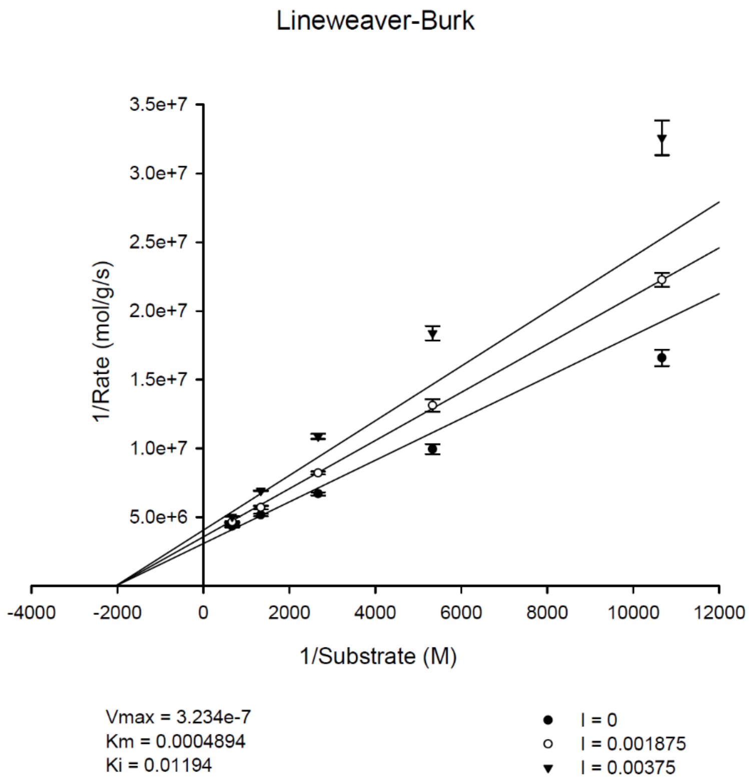
Lineweaver-Burk
1/Rate (mol/g/s)
3.5e+7
3.0e+7
2.5e+7
2.0e+7
1.5e+7
1.0e+7
5.0e+6
-4000
-2000
0
2000
4000
6000
8000
10000
12000
1/Substrate (M)
Vmax = 3.234e-7
Km = 0.0004894
Ki = 0.01194
I = 0
I = 0.001875
I = 0.00375

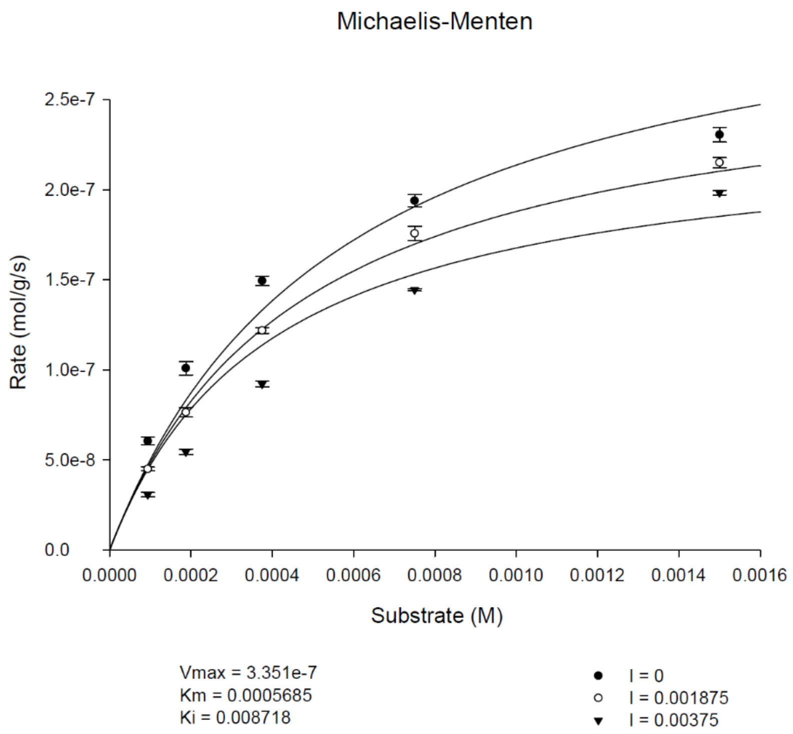
Michaelis-Menten
Rate (mol/g/s)
Substrate (M)
2.5e-7
2.0e-7
1.5e-7
1.0e-7
5.0e-8
0.0
0.0000
0.0002
0.0004
0.0006
0.0008
0.0010
0.0012
0.0014
0.0016
Vmax = 3.351e-7
Km = 0.0005685
Ki = 0.008718
I = 0
I = 0.001875
I = 0.00375

Fig. S2 c)

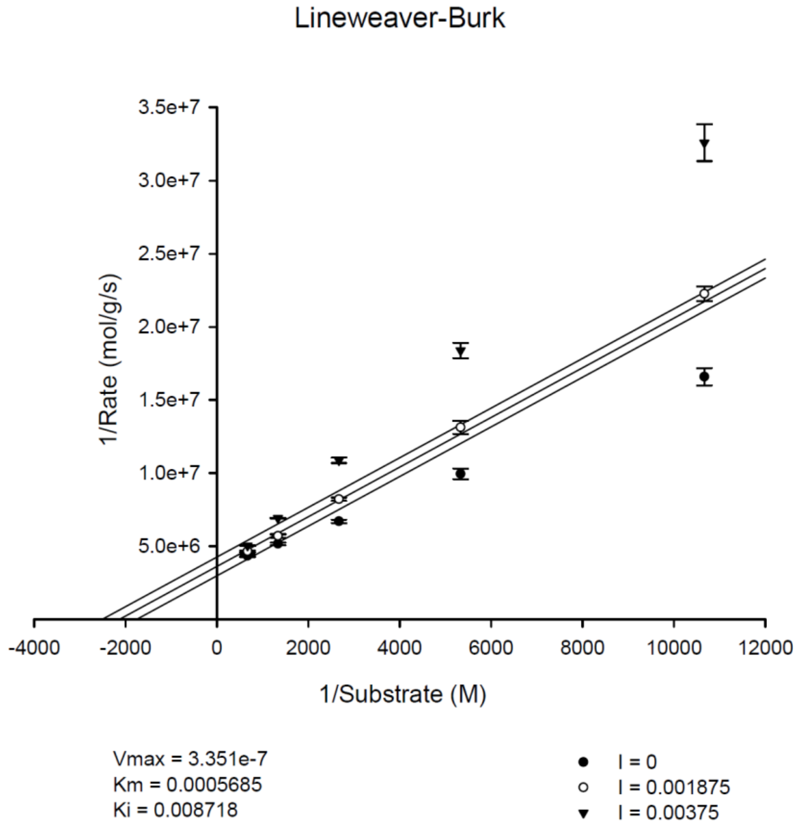
Lineweaver-Burk
1/Rate (mol/g/s)
1/Substrate (M)
3.5e+7
3.0e+7
2.5e+7
2.0e+7
1.5e+7
1.0e+7
5.0e+6
-4000
-2000
0
2000
4000
6000
8000
10000
12000
Vmax = 3.351e-7
Km = 0.0005685
Ki = 0.008718
I = 0
I = 0.001875
I = 0.00375

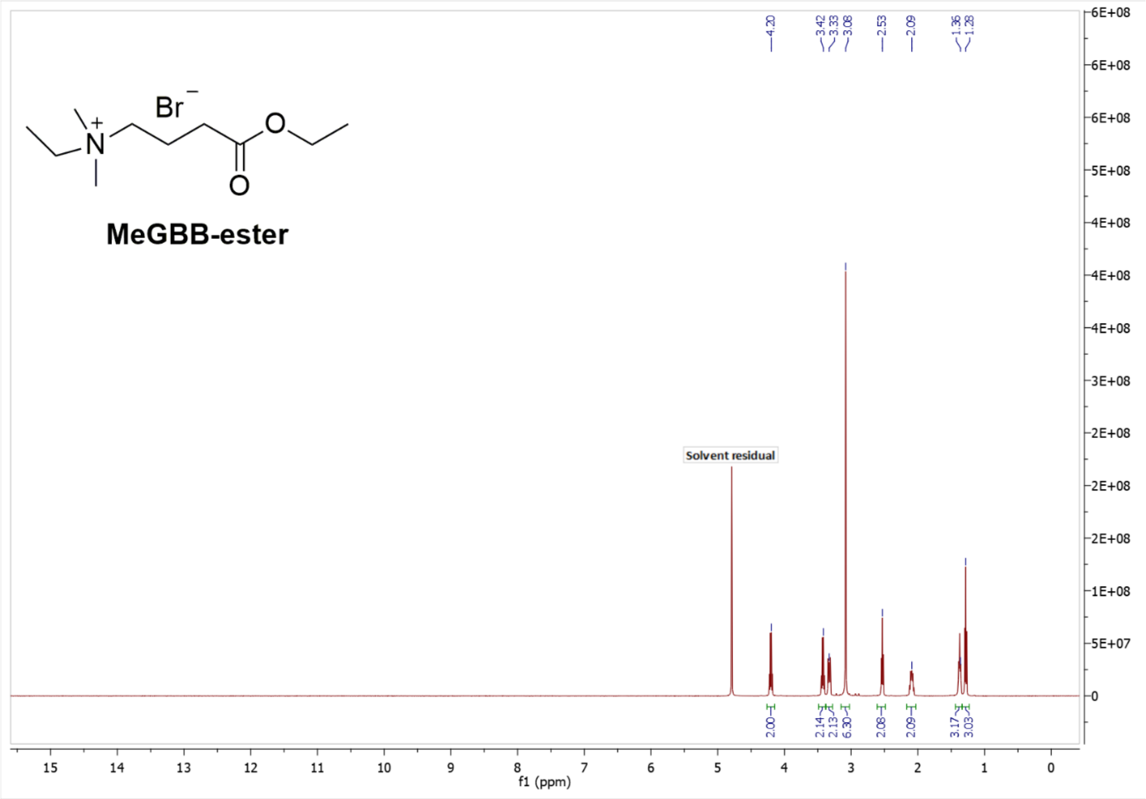

Figure S3. ^{1}H-NMR Spectrum of MeGBB-ester D_2O-d_2.

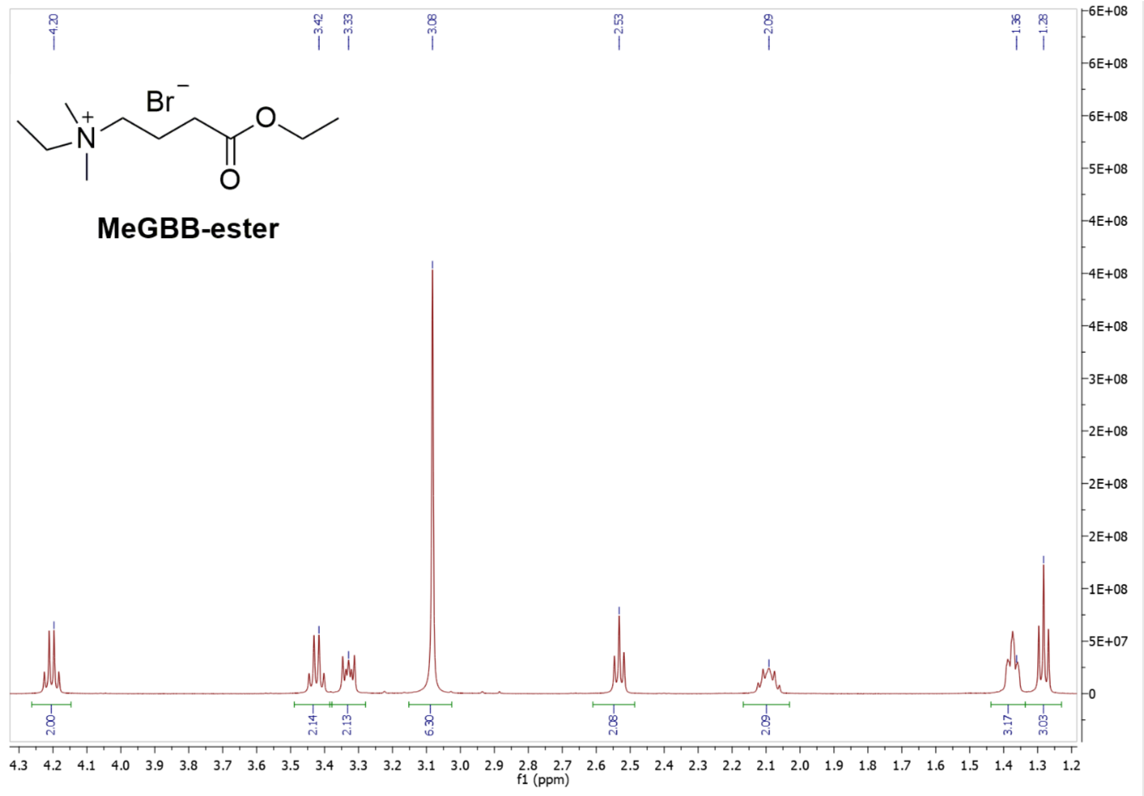

Figure S3a. ^{1}H-NMR Spectrum of MeGBB-ester D_2O-d_2.

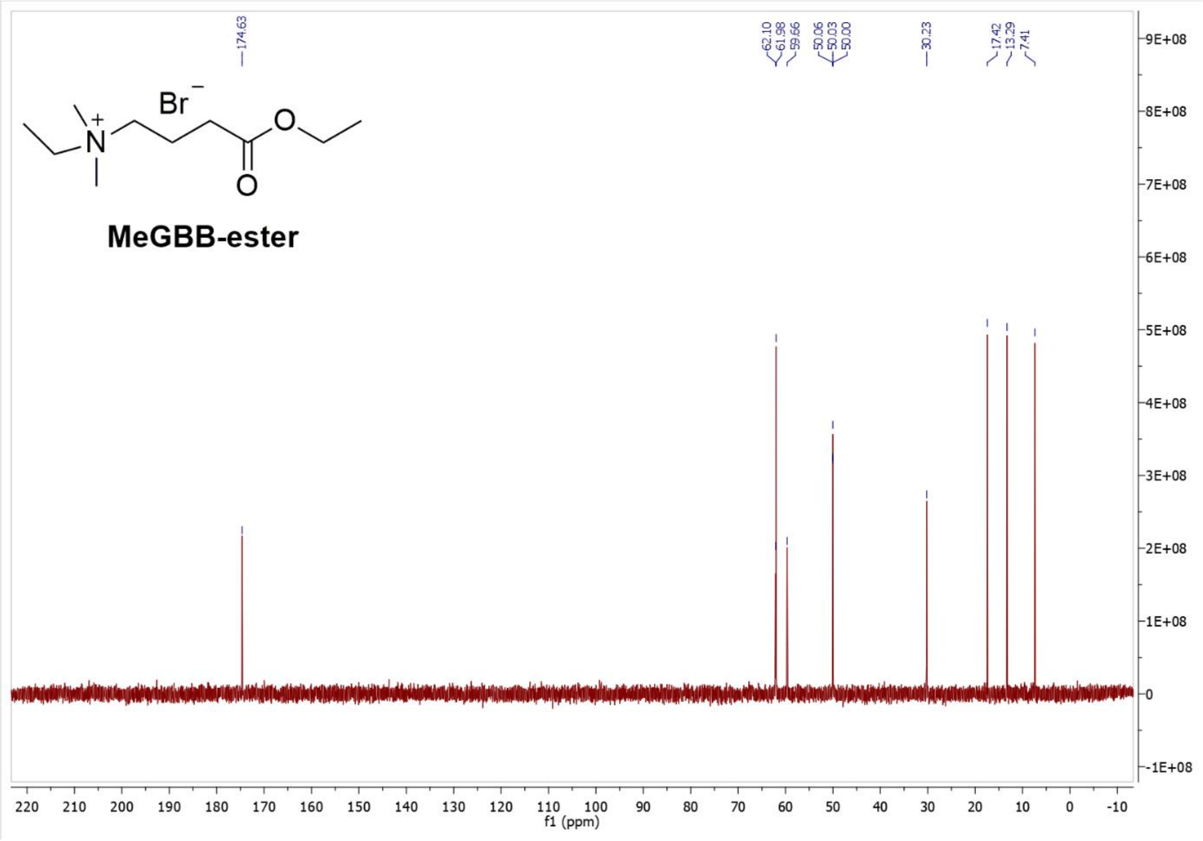

Figure S4. **^{13}C-NMR Spectrum of MeGBB-ester in D_2O-d_2.**

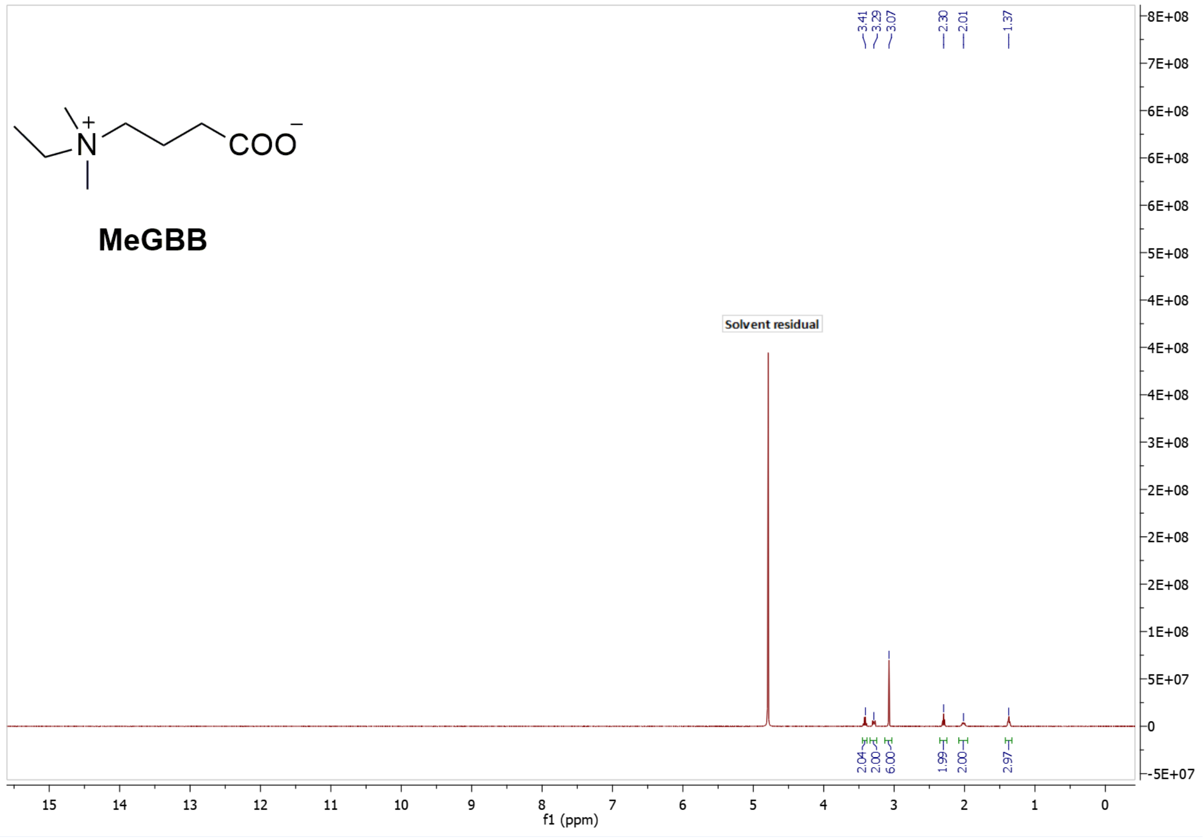

Figure S5. **^{1}H-NMR Spectrum of MeGBB in D_2O-d_2.**

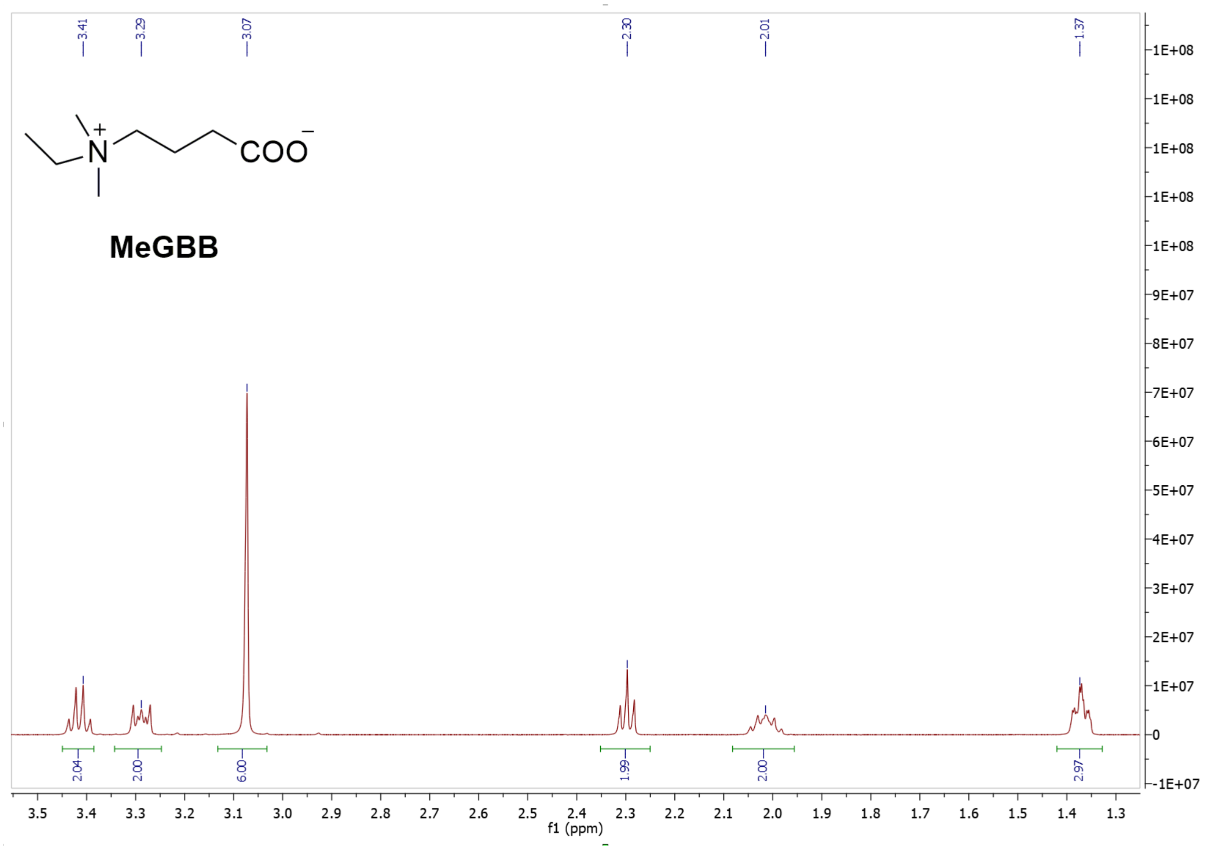

Figure S5a. ¹H-NMR Spectrum of MeGBB in D_2O-d_2.

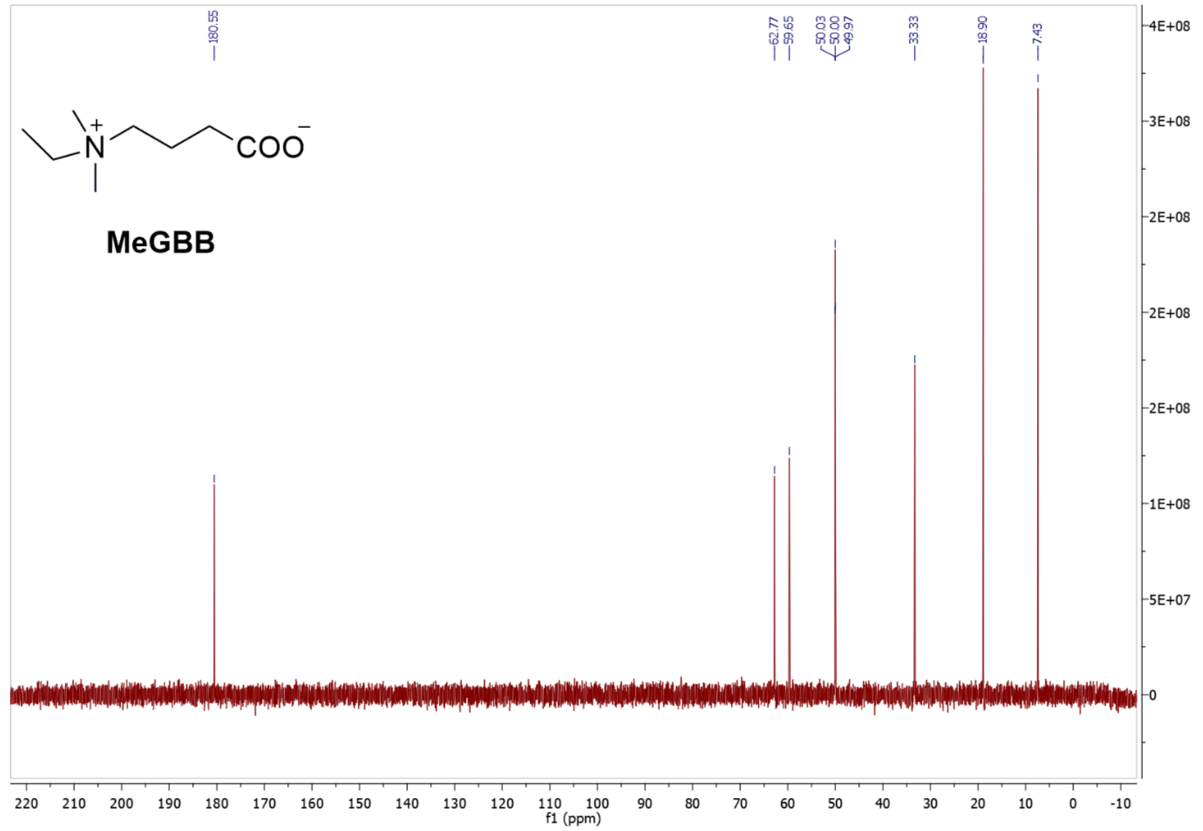

Figure S6. ¹³C-NMR Spectrum of MeGBB in D_2O-d_2.

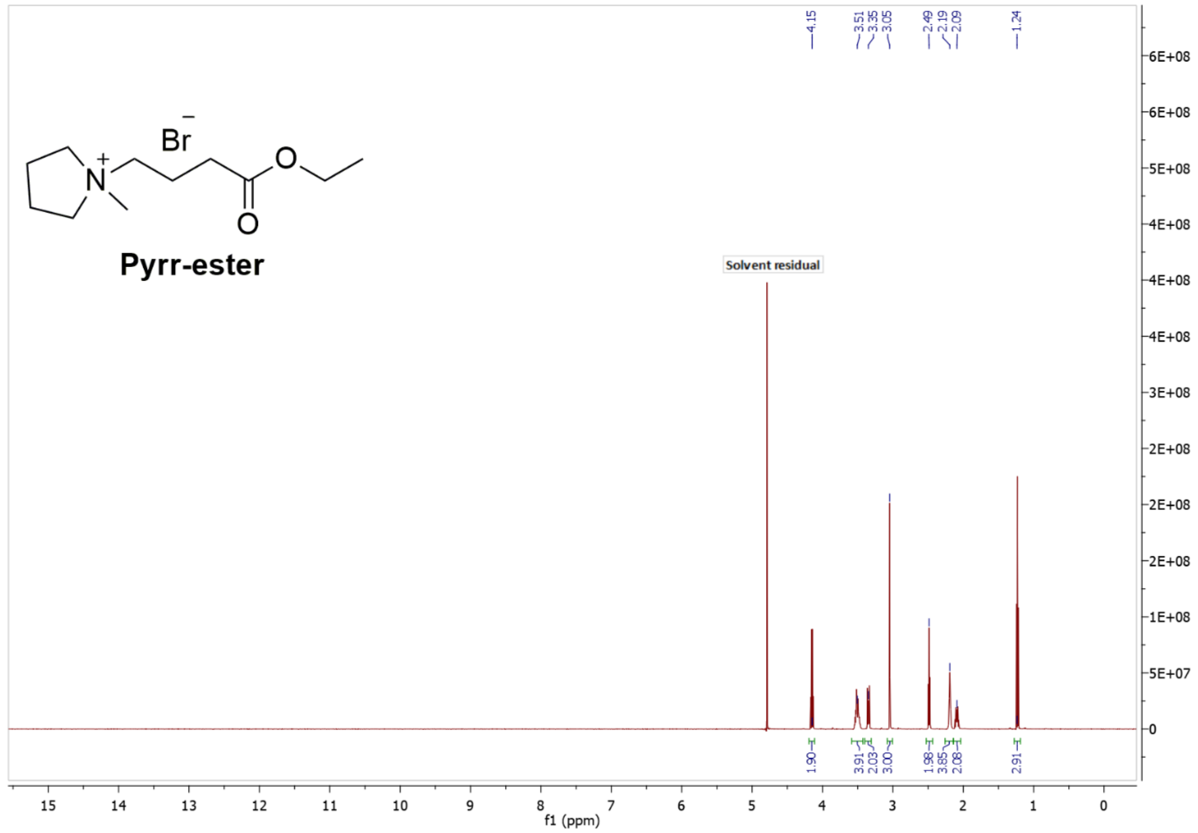

Figure S7. ^{1}**H-NMR Spectrum of Pyrr-ester in** D_2O**-**d_2**.**

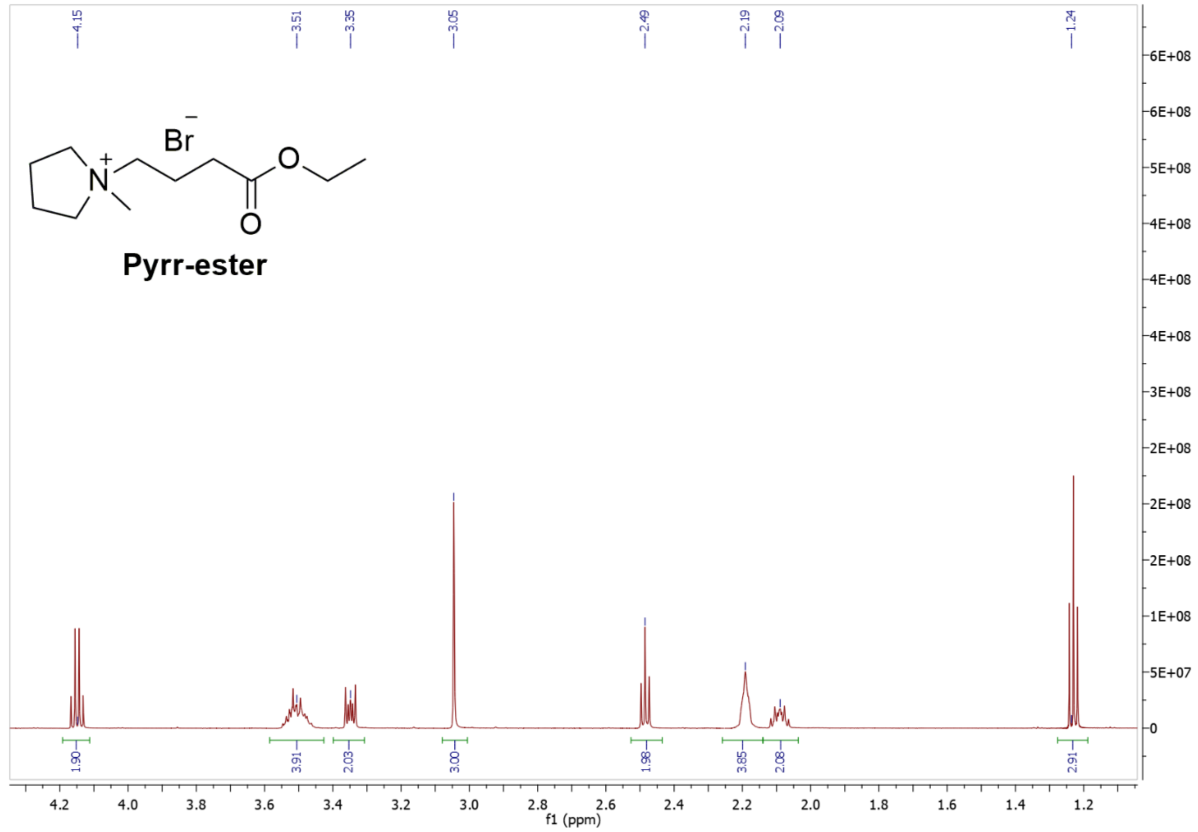

Figure S7a. ^{1}**H-NMR Spectrum of Pyrr-ester in** D_2O**-**d_2**.**

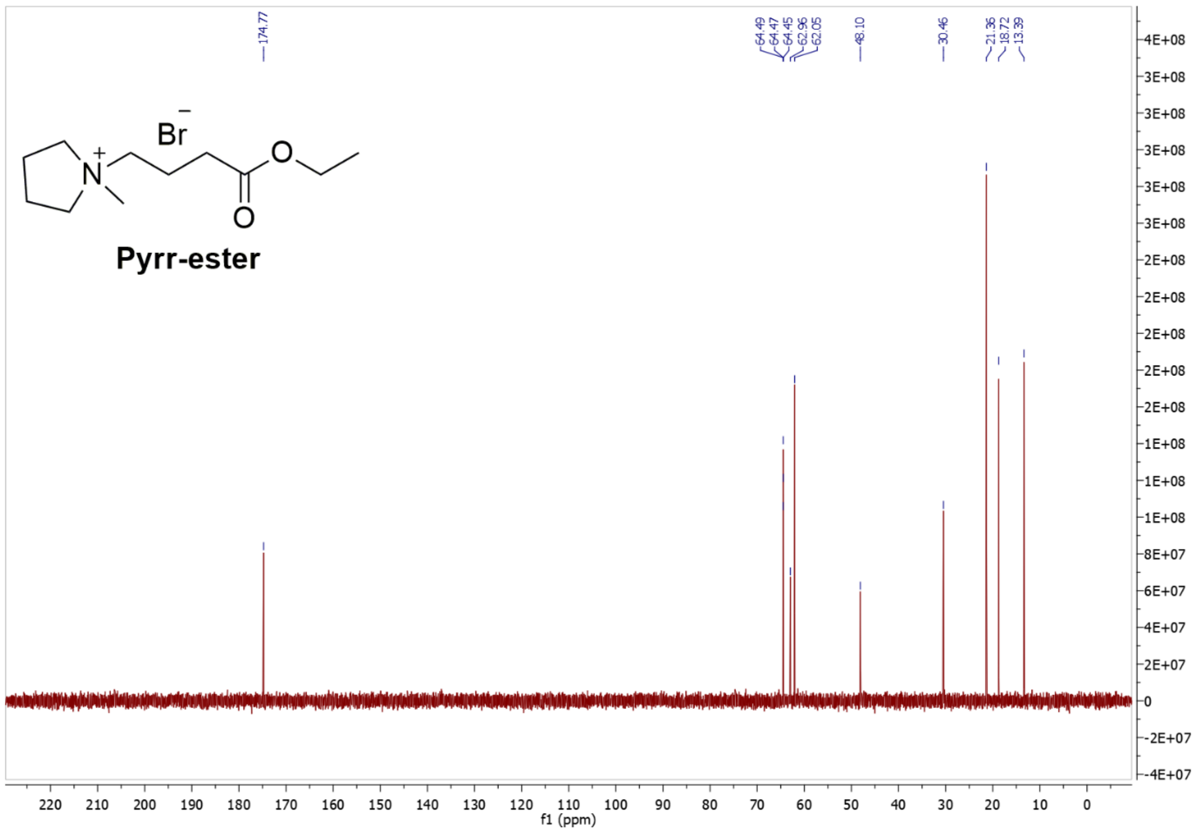

Figure S8. **^{13}C-NMR Spectrum of Pyrr-ester in D_2O-d_2.**

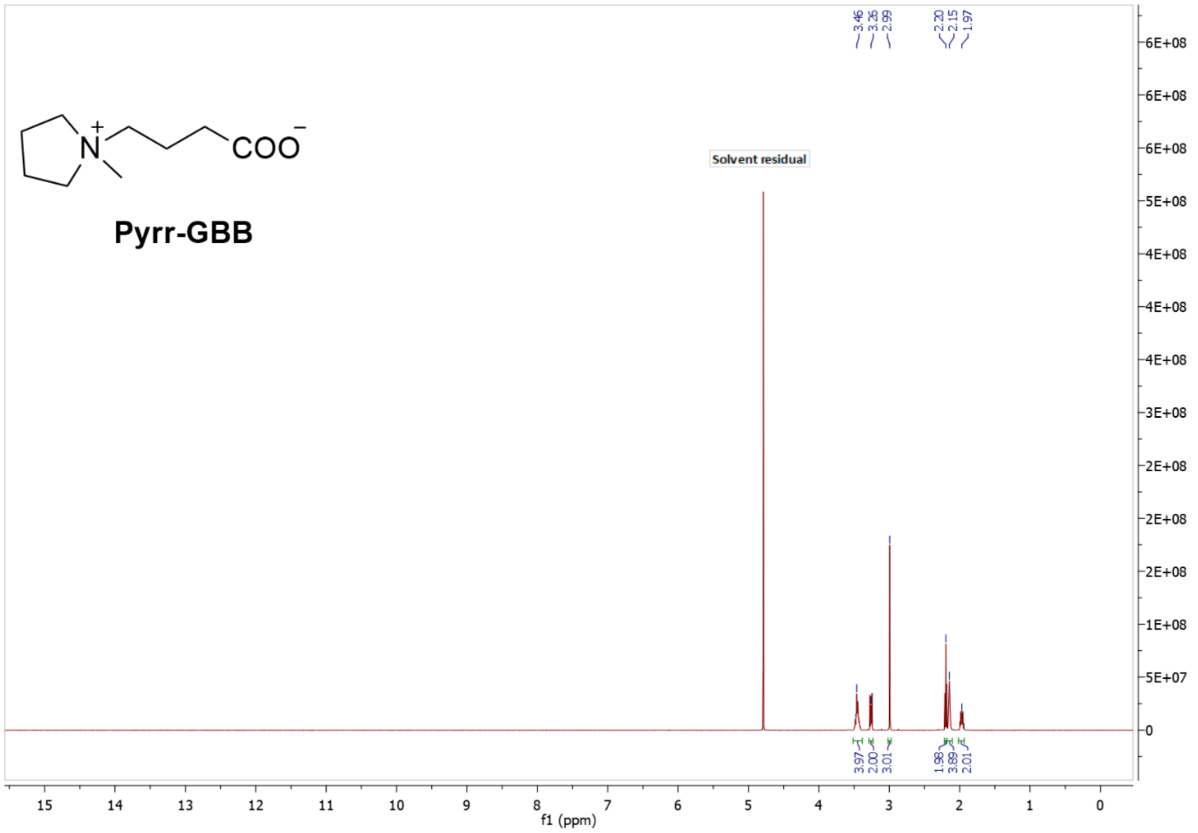

Figure S9. **^{1}H-NMR Spectrum of Pyrr-GBB in D_2O-d_2.**

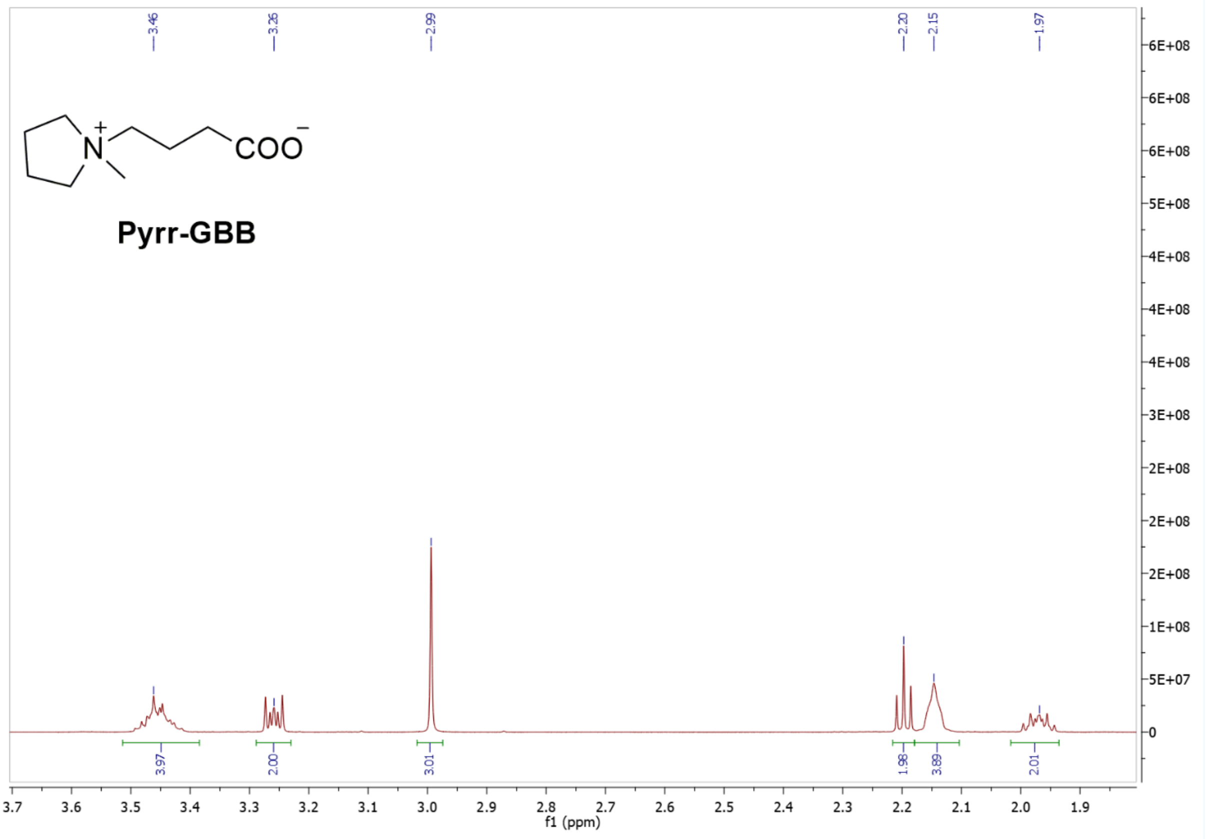

Figure S9a. **^{1}H-NMR Spectrum of Pyrr-GBB in D_2O-d_2.**

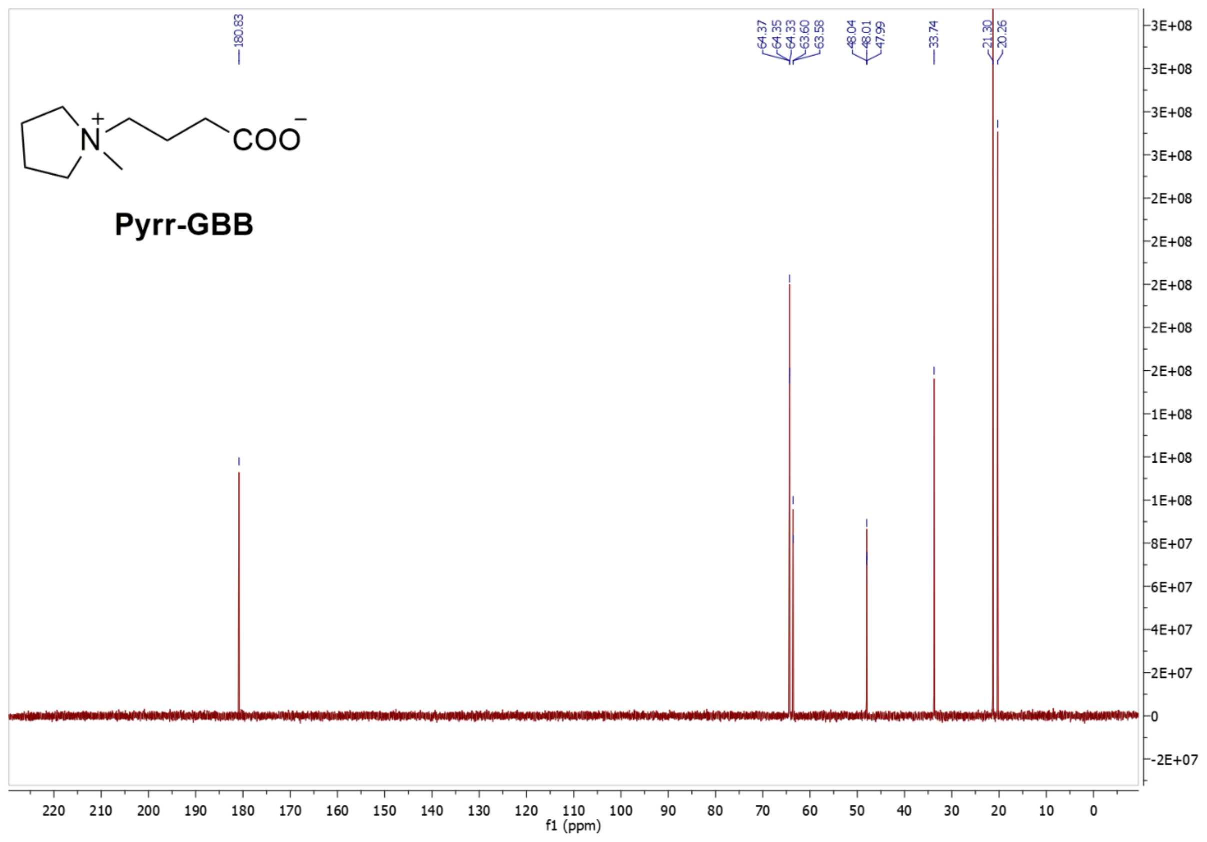

Figure S10. **^{13}C-NMR Spectrum of Pyrr-GBB in D_2O-d_2.**

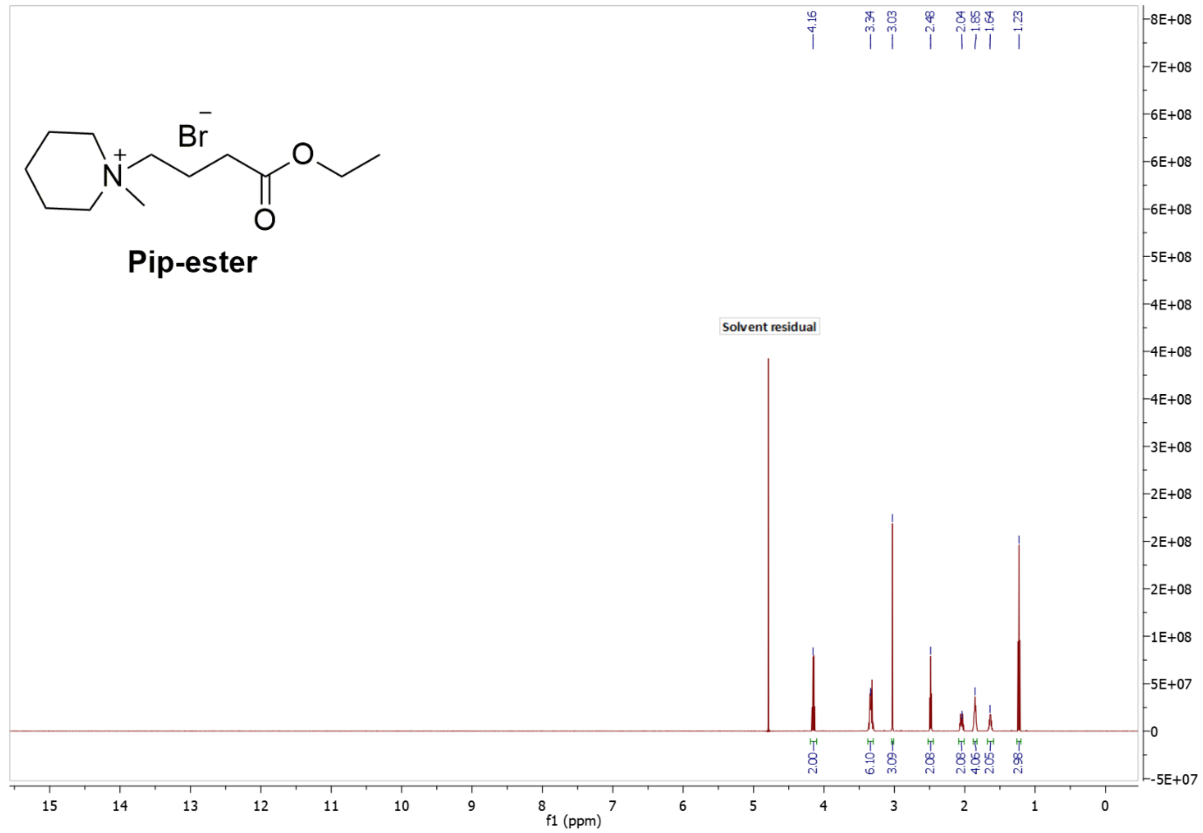

Figure S11. **^{1}H-NMR Spectrum of Pip-ester in D_2O-d2.**

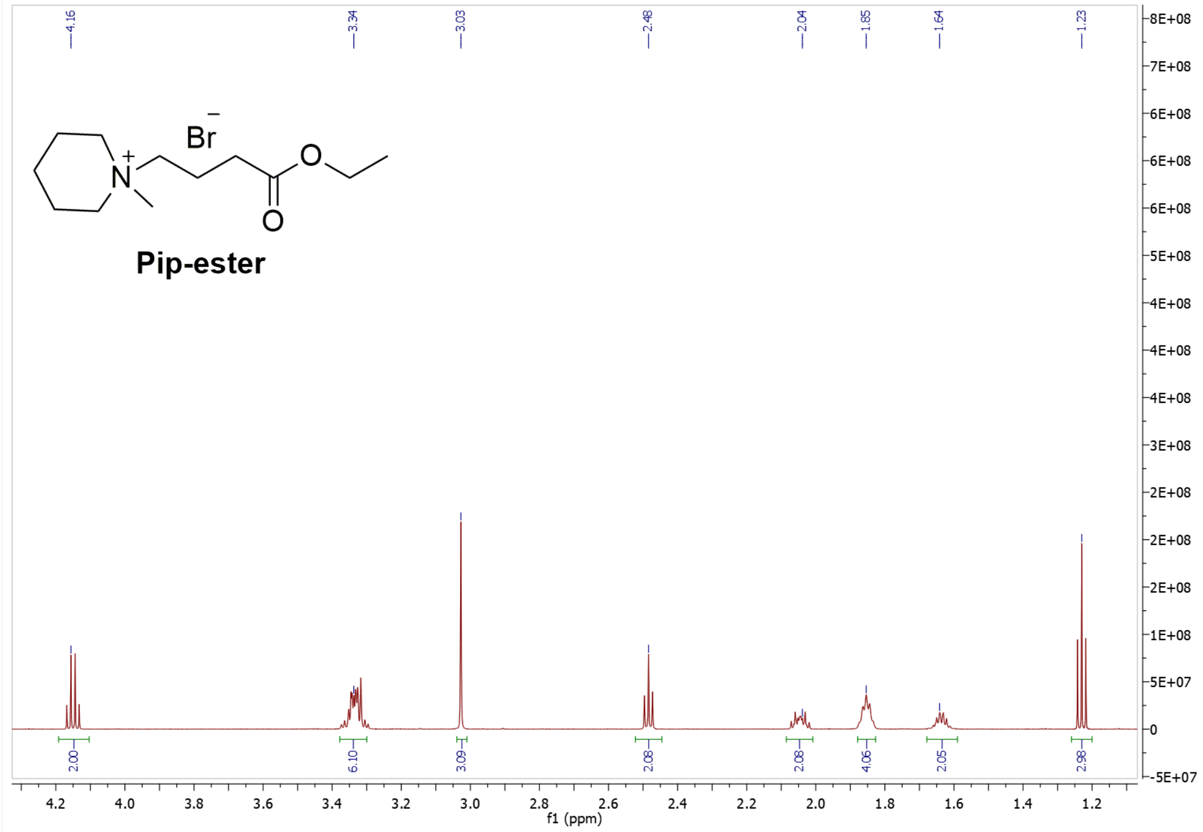

Figure S11a. **^{1}H-NMR Spectrum of Pip-ester in D_2O-d2.**

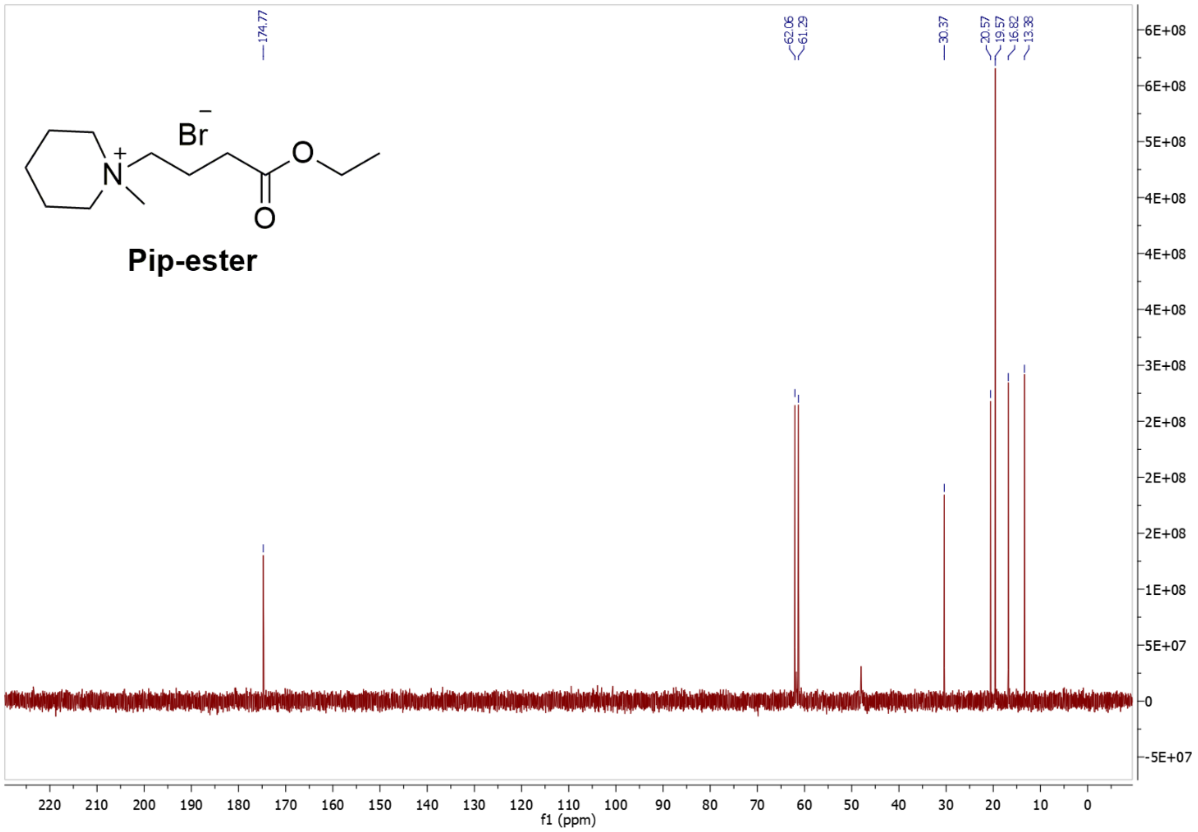

Figure S12. **^{13}C-NMR Spectrum of Pip-ester in D_2O-d_2.**

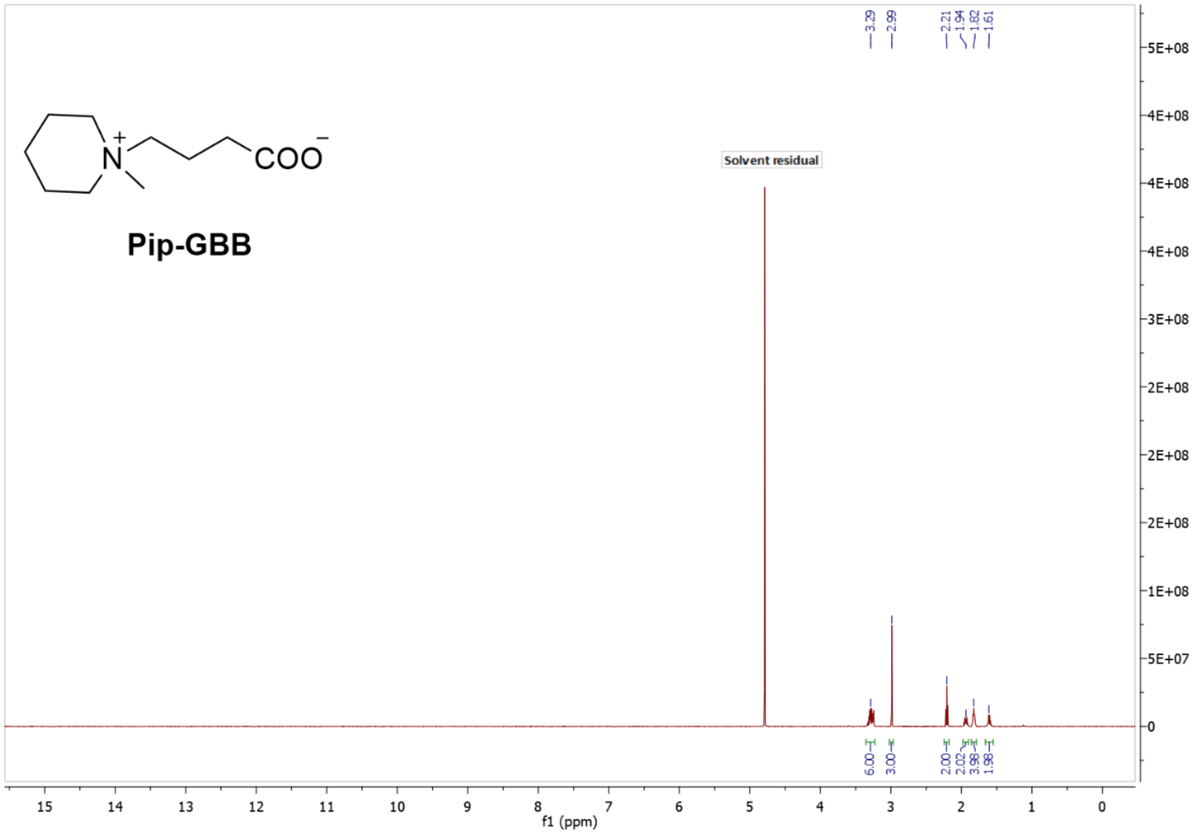

Figure S13. **^{1}H-NMR Spectrum of Pip-GBB in D_2O-d_2.**

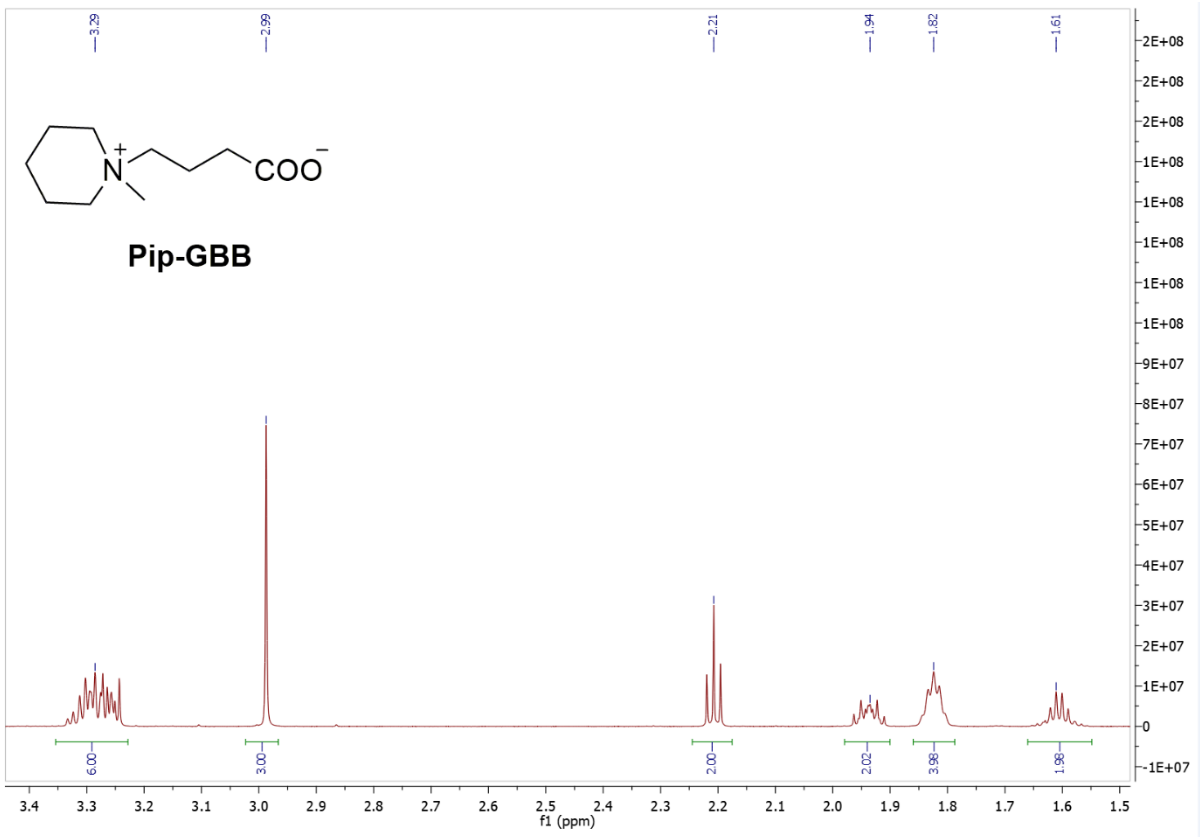

Figure S13a. ^{1}**H-NMR Spectrum of Pip-GBB in** D_2O**-**d_2**.**

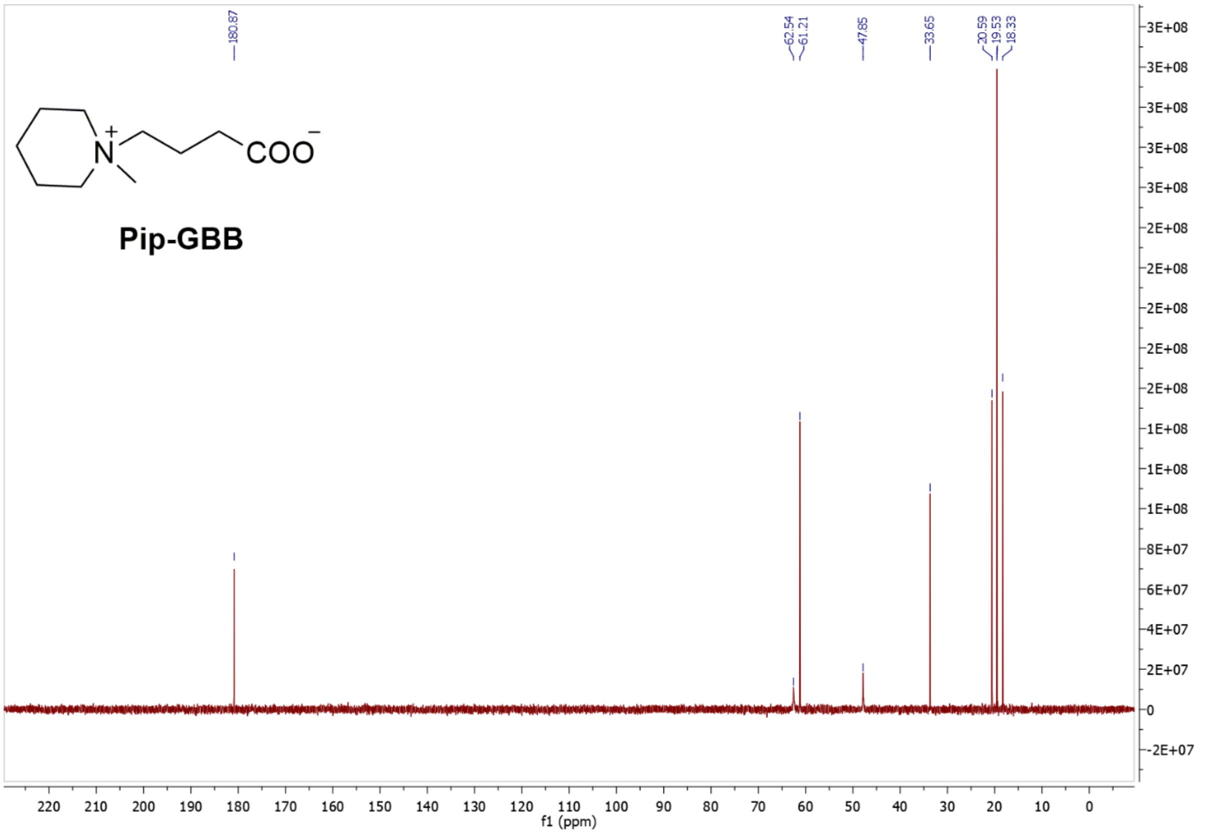

Figure S14. ^{13}**C-NMR Spectrum of Pip-GBB in** D_2O**-**d_2**.**

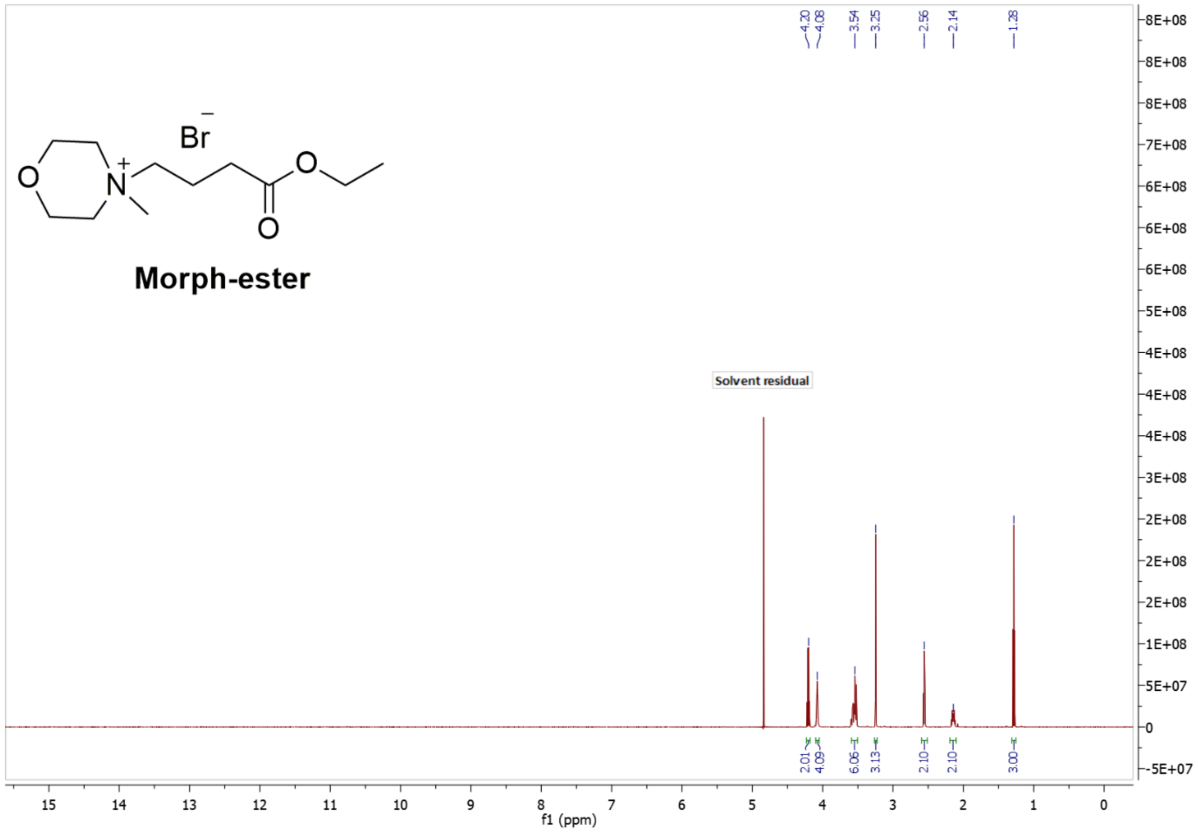

Figure S15. ¹H-NMR Spectrum of Morph-ester in D_2O-d_2.

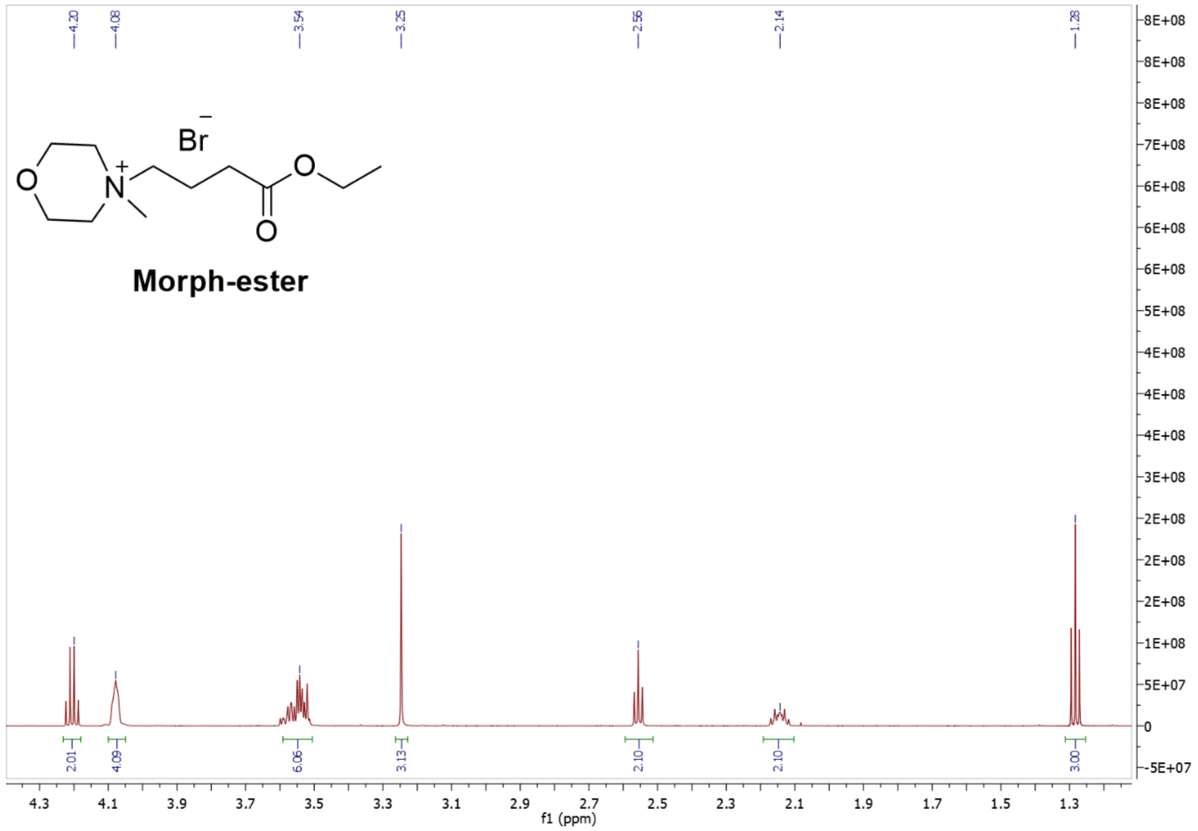

Figure S15a. ¹H-NMR Spectrum of Morph-ester in D_2O-d_2.

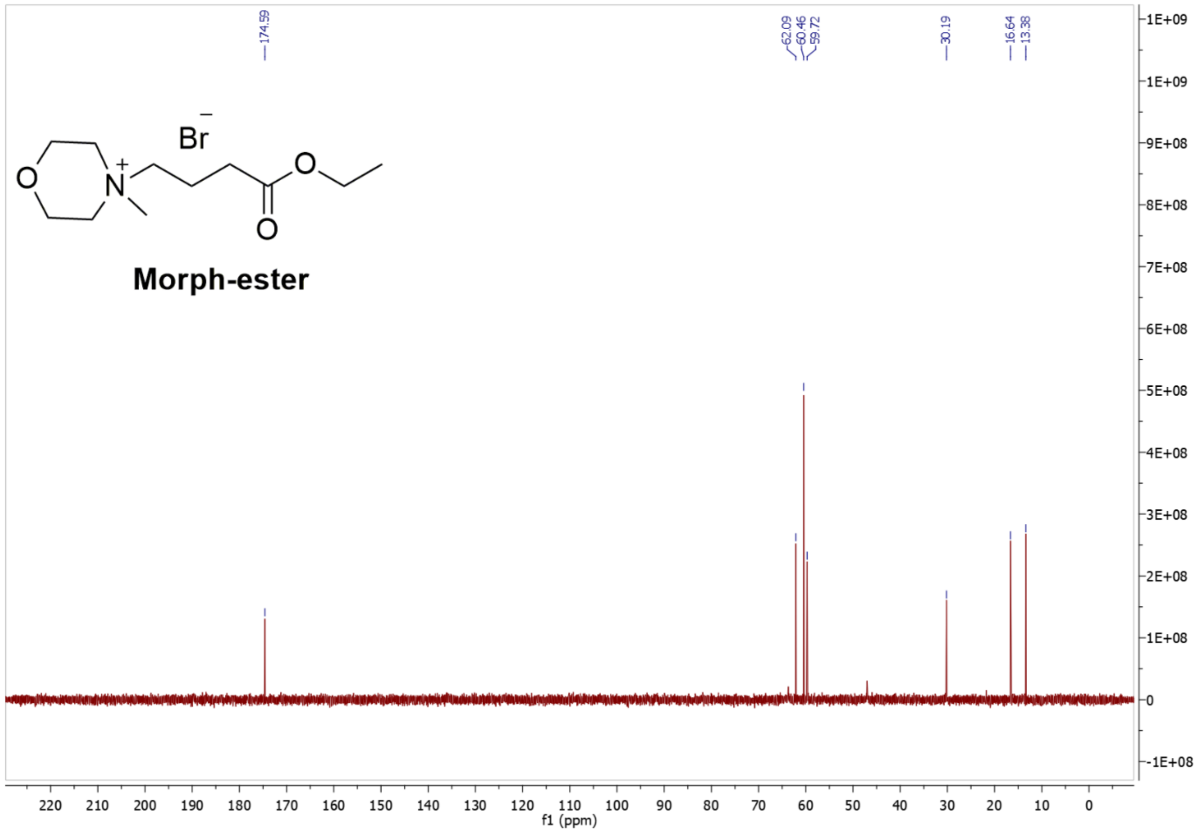

Figure S16. ^{13}**C-NMR Spectrum of Morph-ester in** D_2O**-**d_2**.**

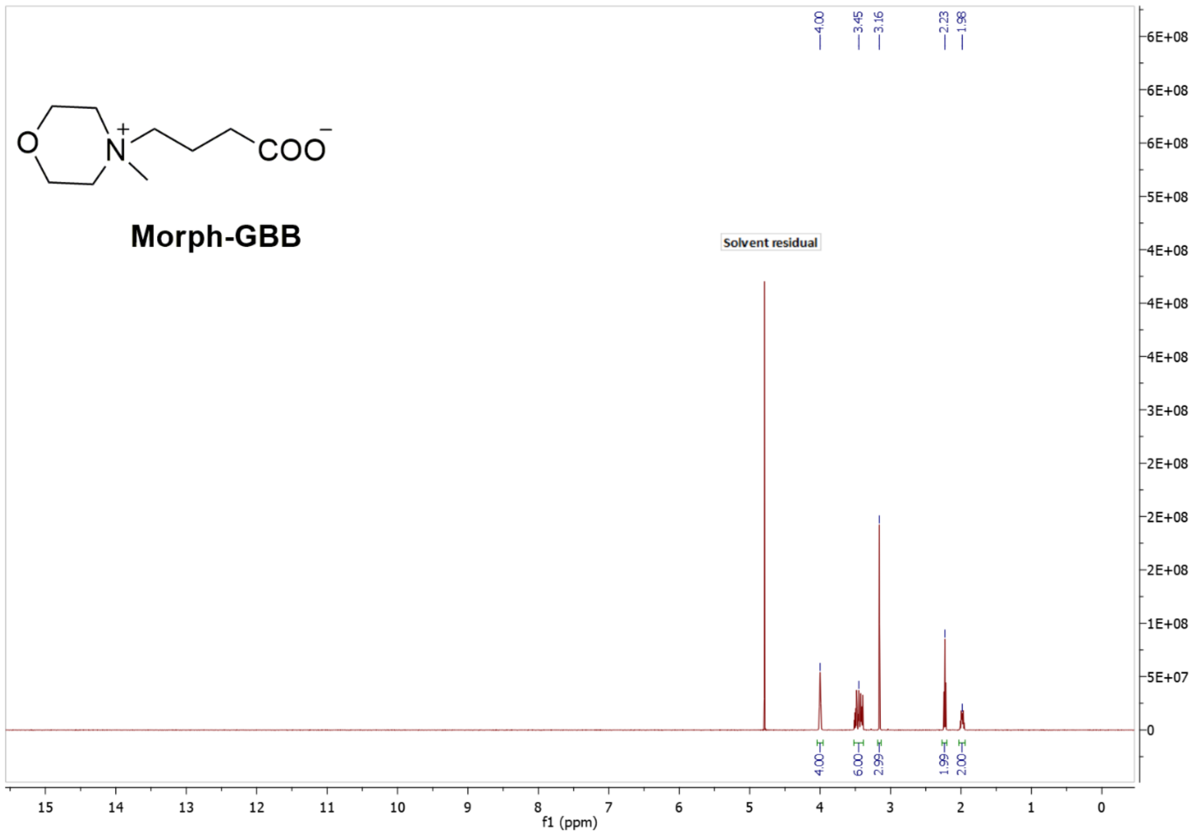

Figure S17. ^{1}**H-NMR Spectrum of Morph-GBB in** D_2O**-**d_2**.**

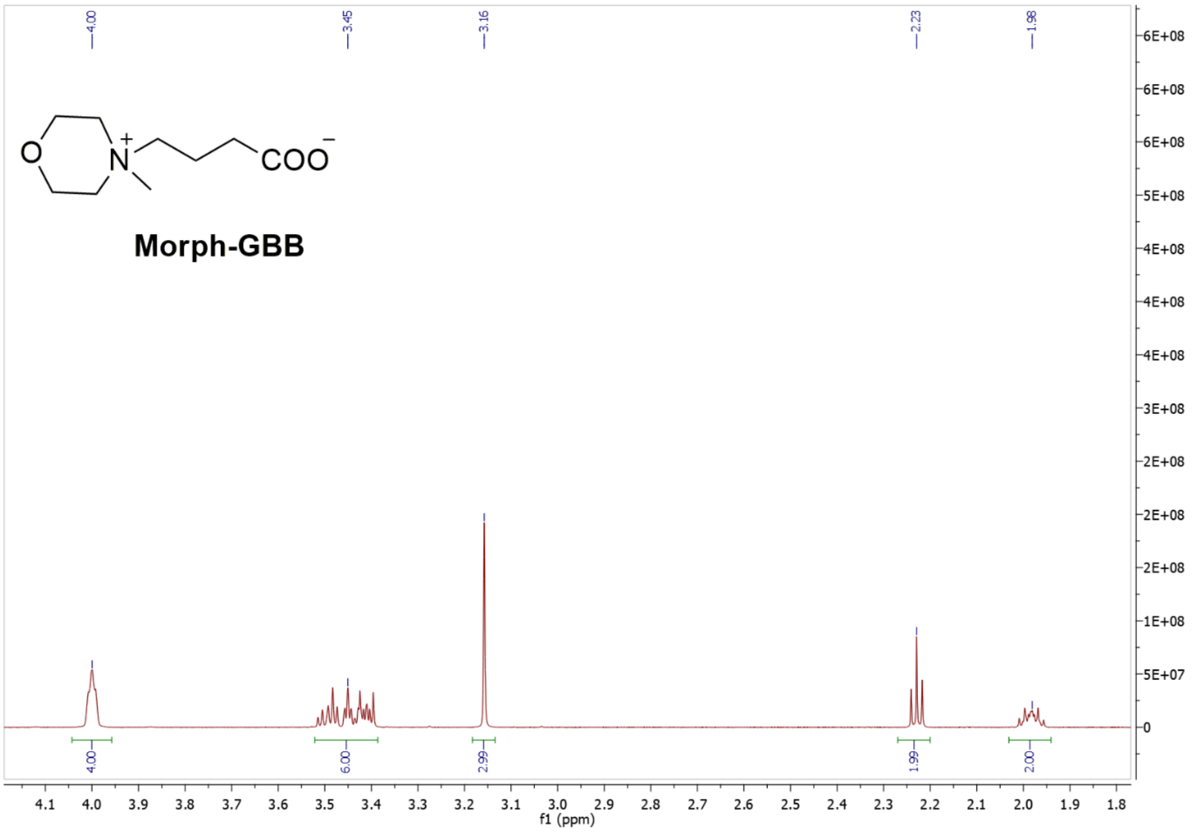

Figure S17a. **¹H-NMR Spectrum of Morph-GBB in D₂O-d₂.**

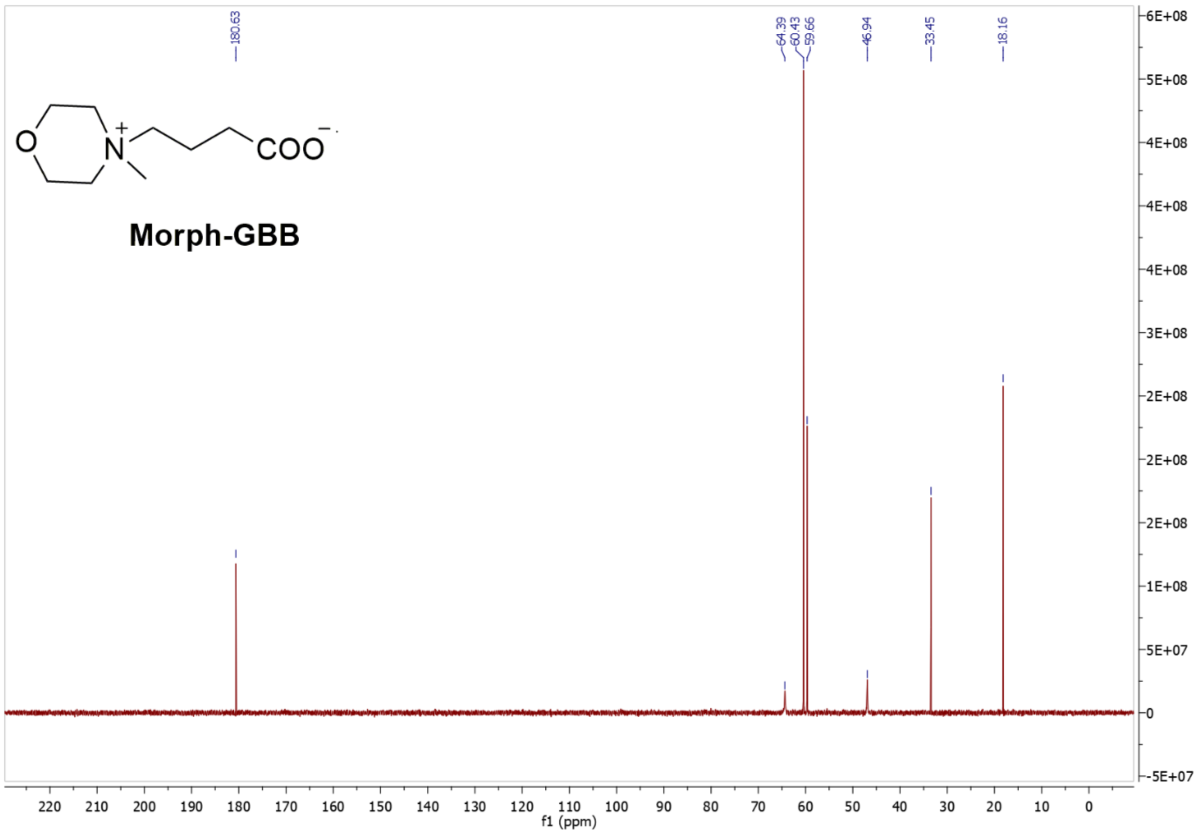

Figure S18. **¹³C-NMR Spectrum of Morph-GBB in D₂O-d₂.**

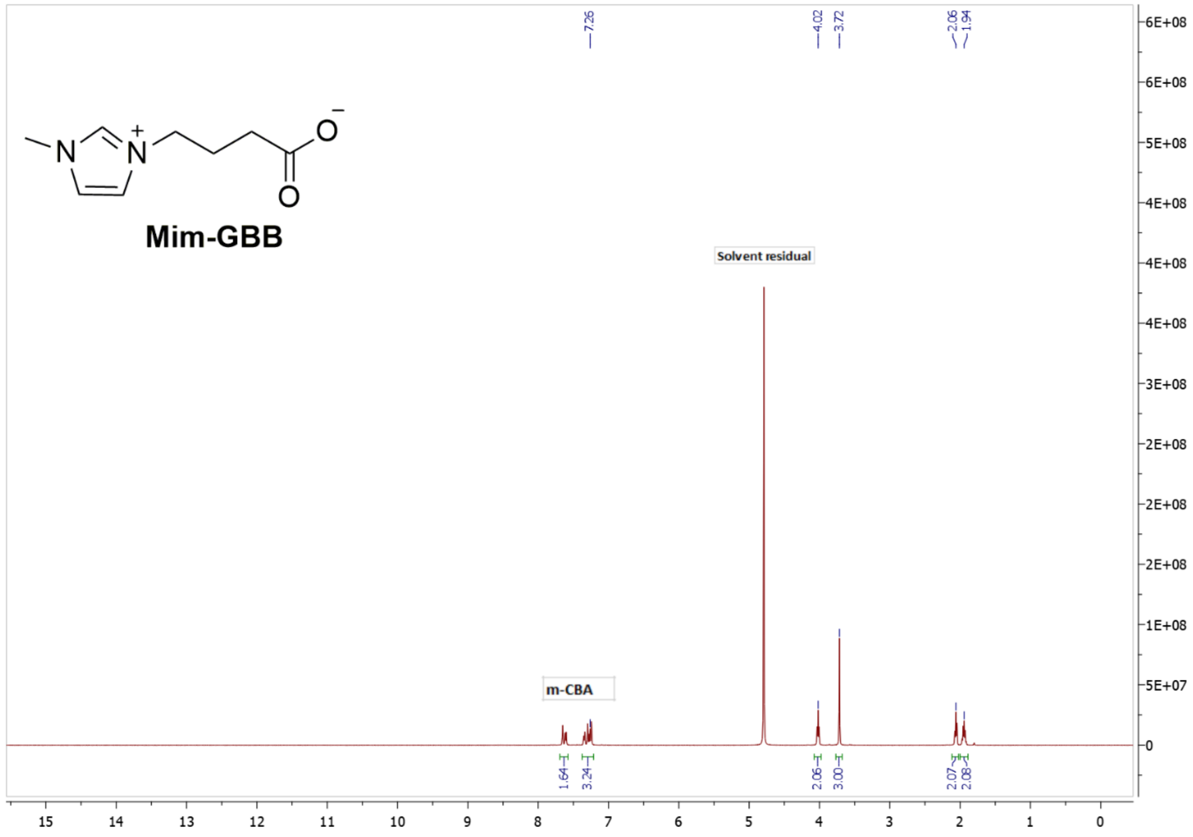

Figure S19. ^{1}H-NMR Spectrum of Mim-GBB in D_2O-d_2.

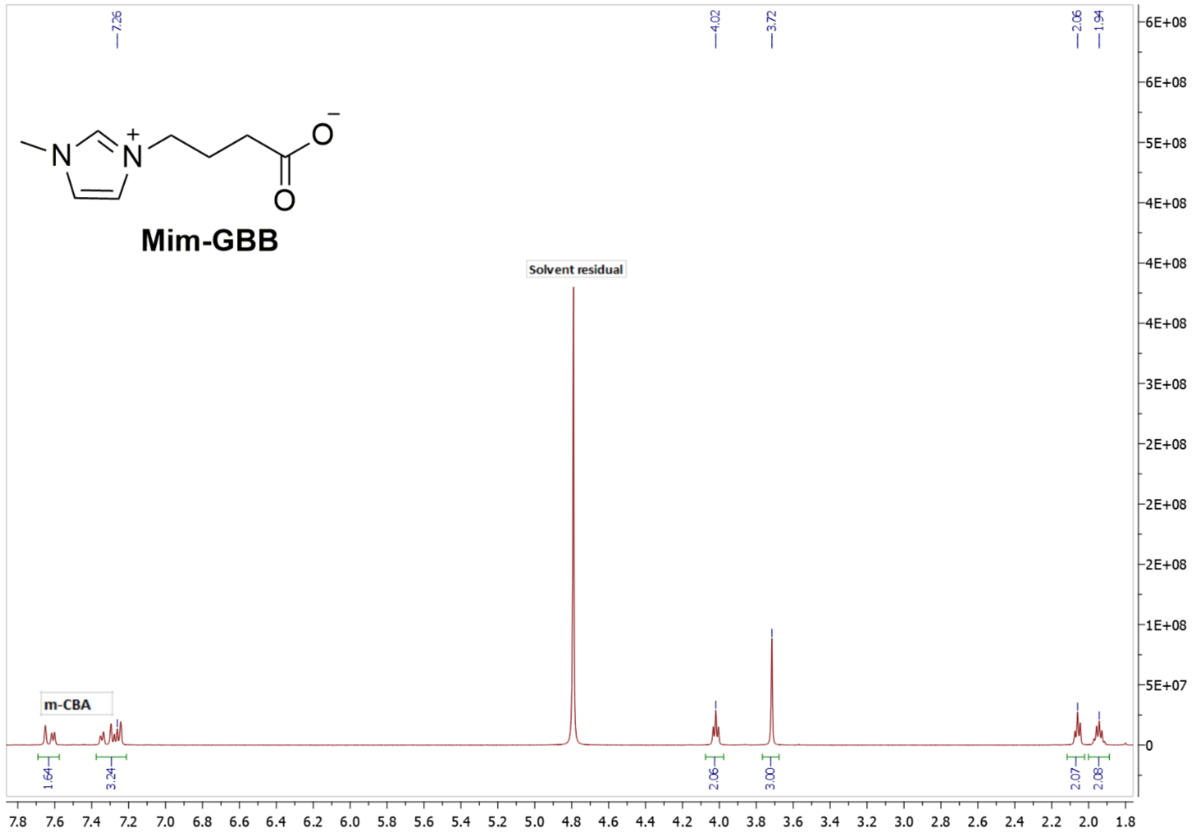

Figure S19a. ^{1}H-NMR Spectrum of Mim-GBB in D_2O-d_2.

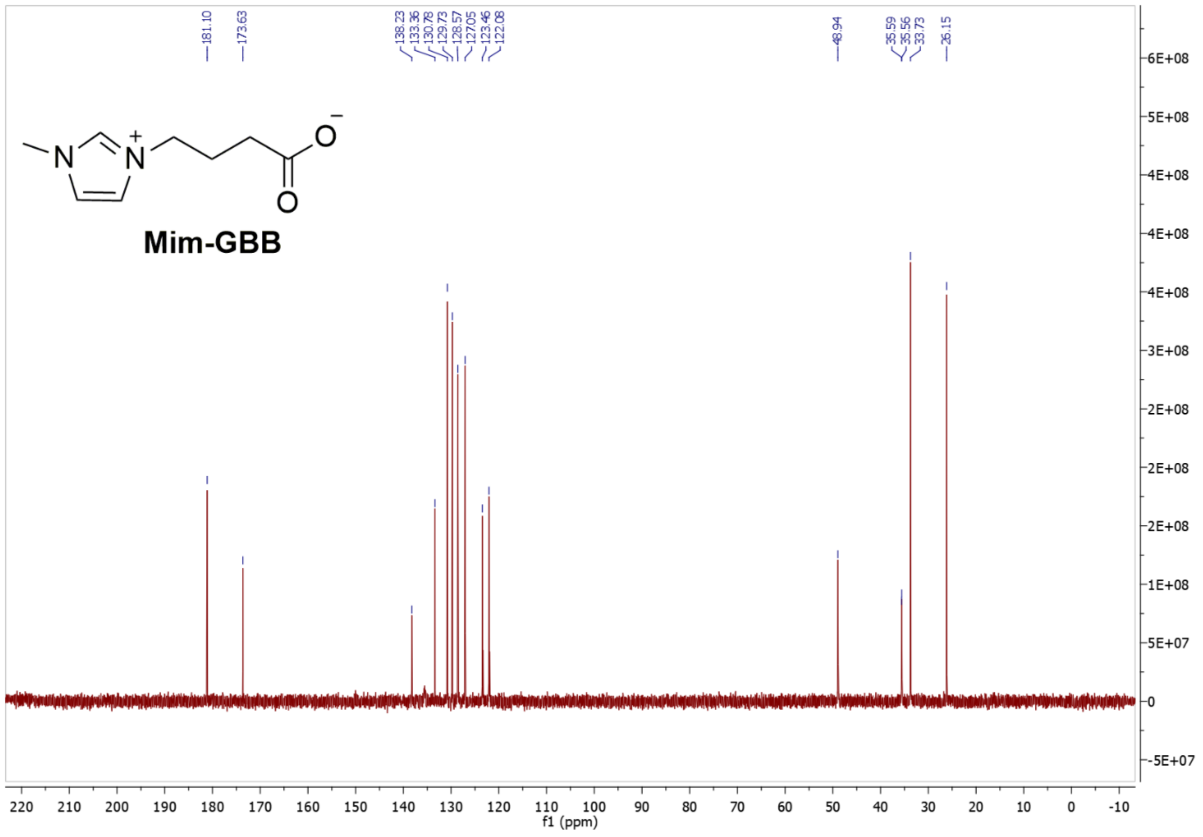

Figure S20. ^{13}**C-NMR Spectrum of Mim-GBB in** D_2O**-d**$_2$**.**

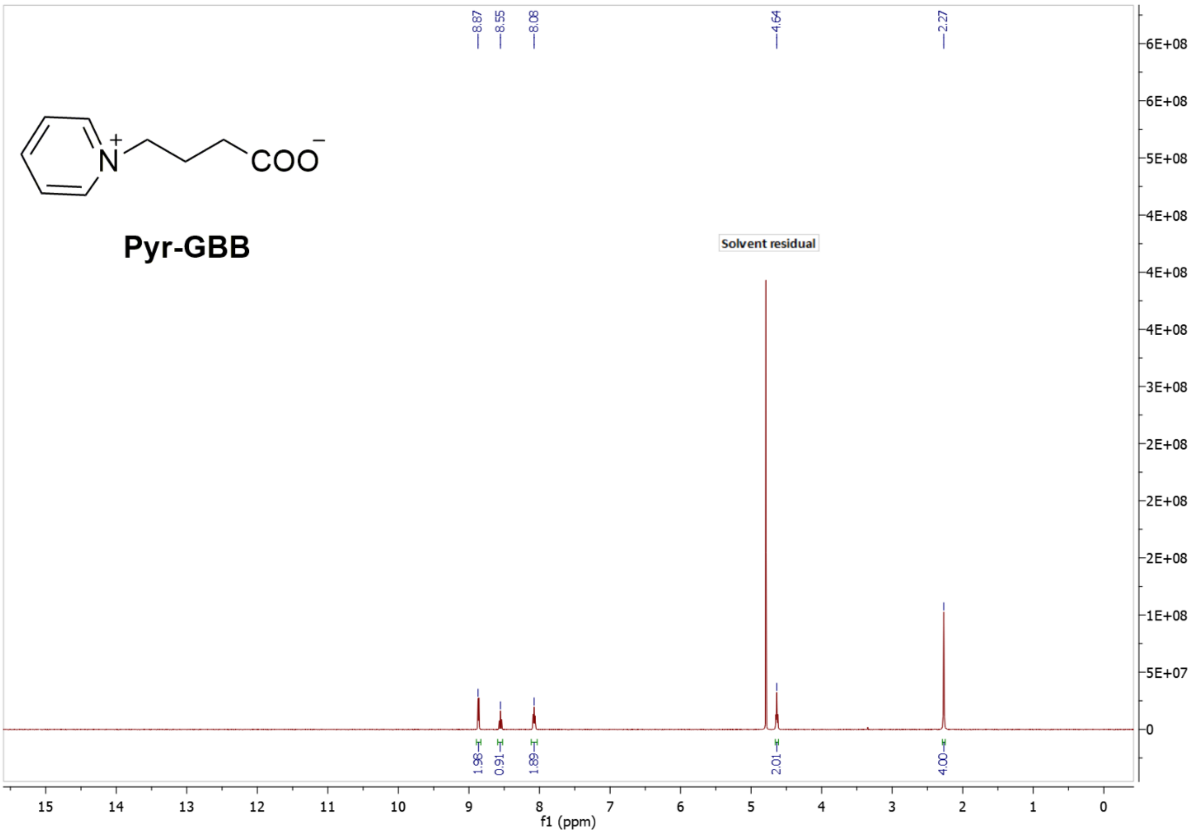

Figure S21. ^{1}**H-NMR Spectrum of Pyr-GBB in** D_2O**-d**$_2$**.**

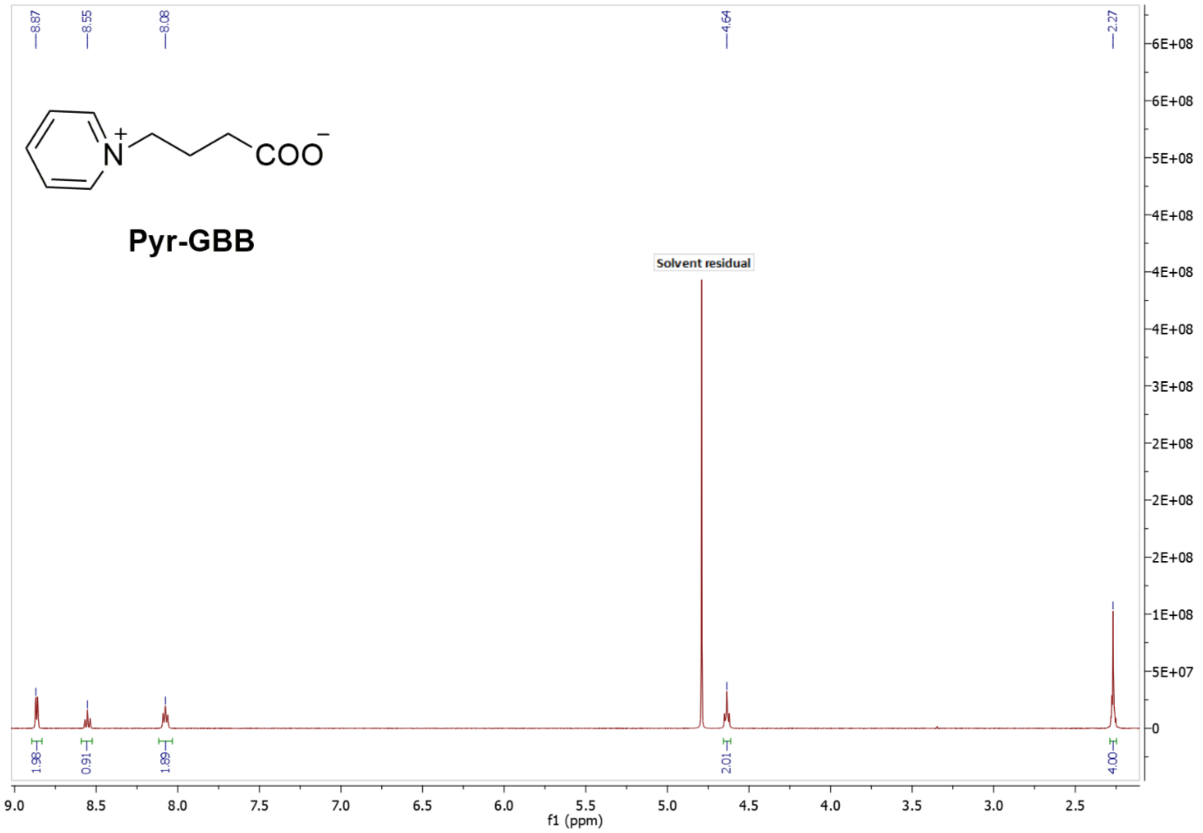

Figure S21a. ^{1}H-NMR Spectrum of Pyr-GBB in D_2O-d$_2$.

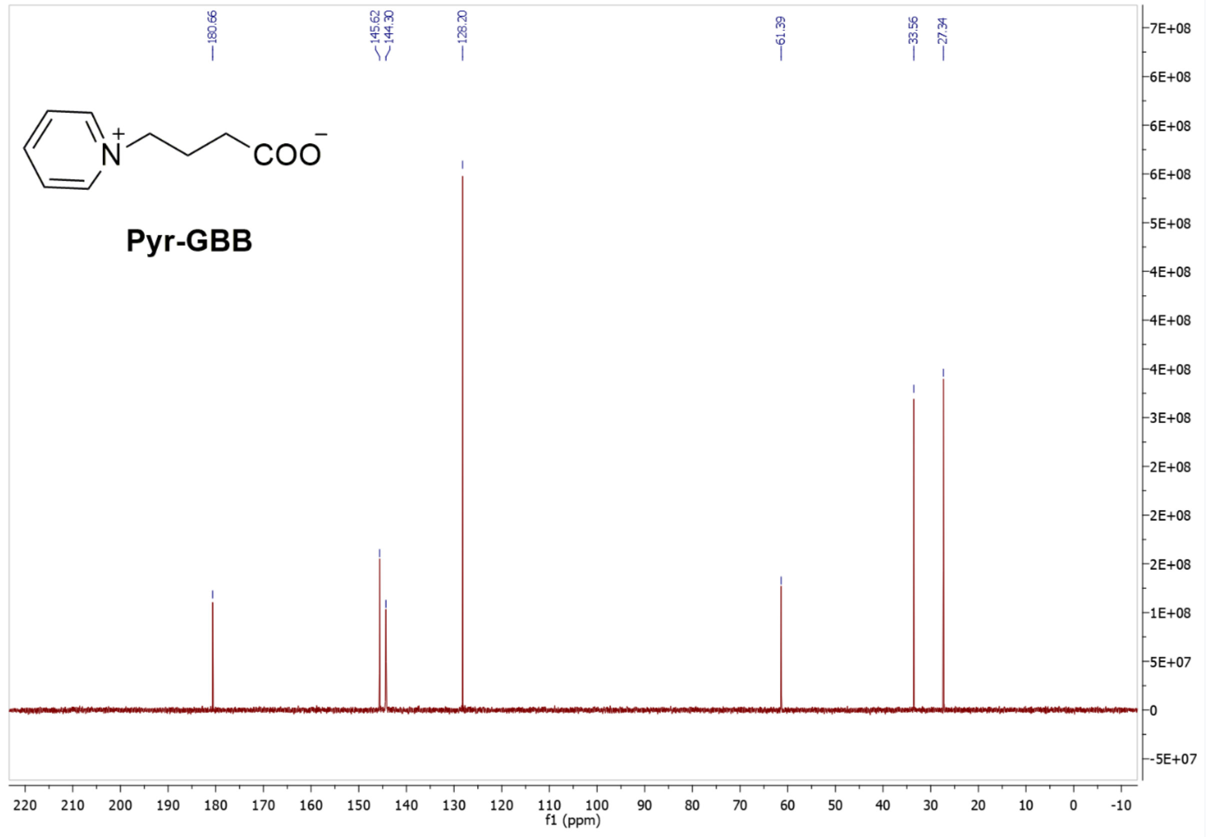

Figure S22. ^{13}C-NMR Spectrum of Pyr-GBB in D_2O-d$_2$.

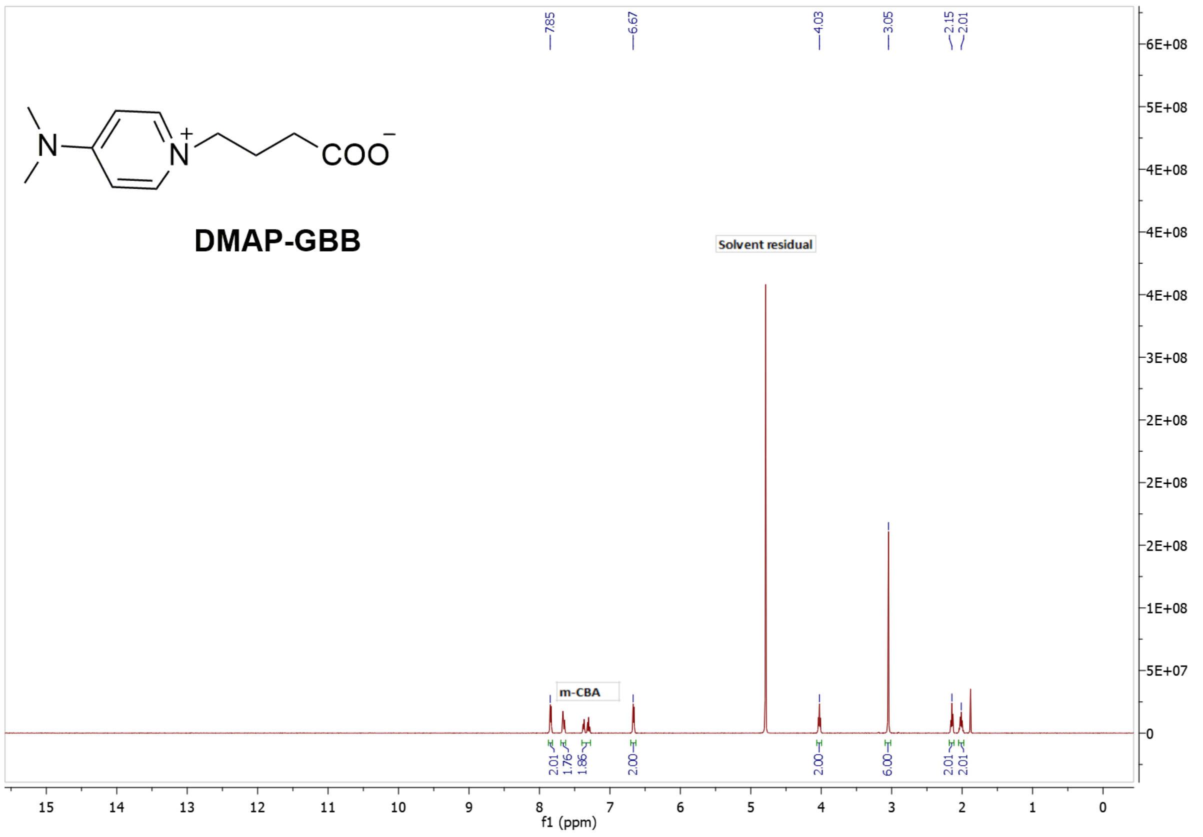

Figure S23. ^{1}**H-NMR Spectrum of DMAP-GBB in** D_2O**-d**$_2$**.**

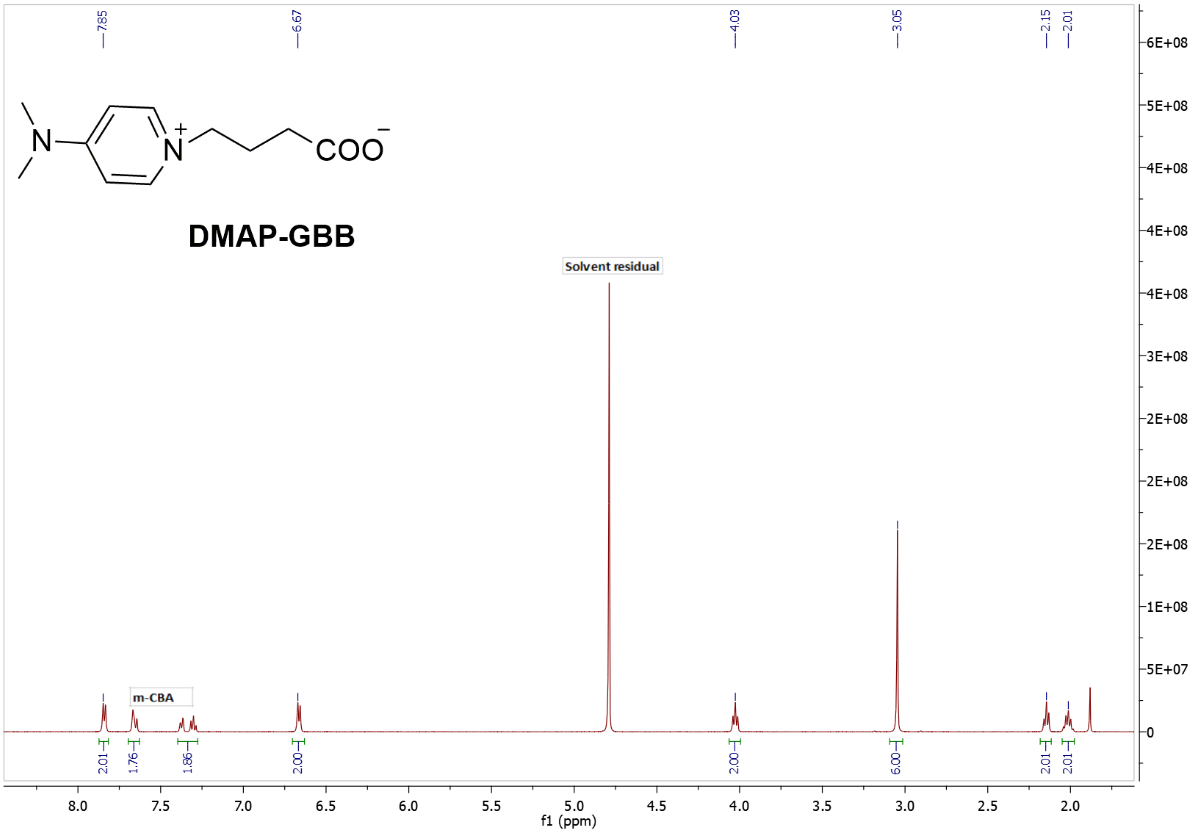

Figure S23a. ^{1}**H-NMR Spectrum of DMAP-GBB in** D_2O**-d**$_2$**.**

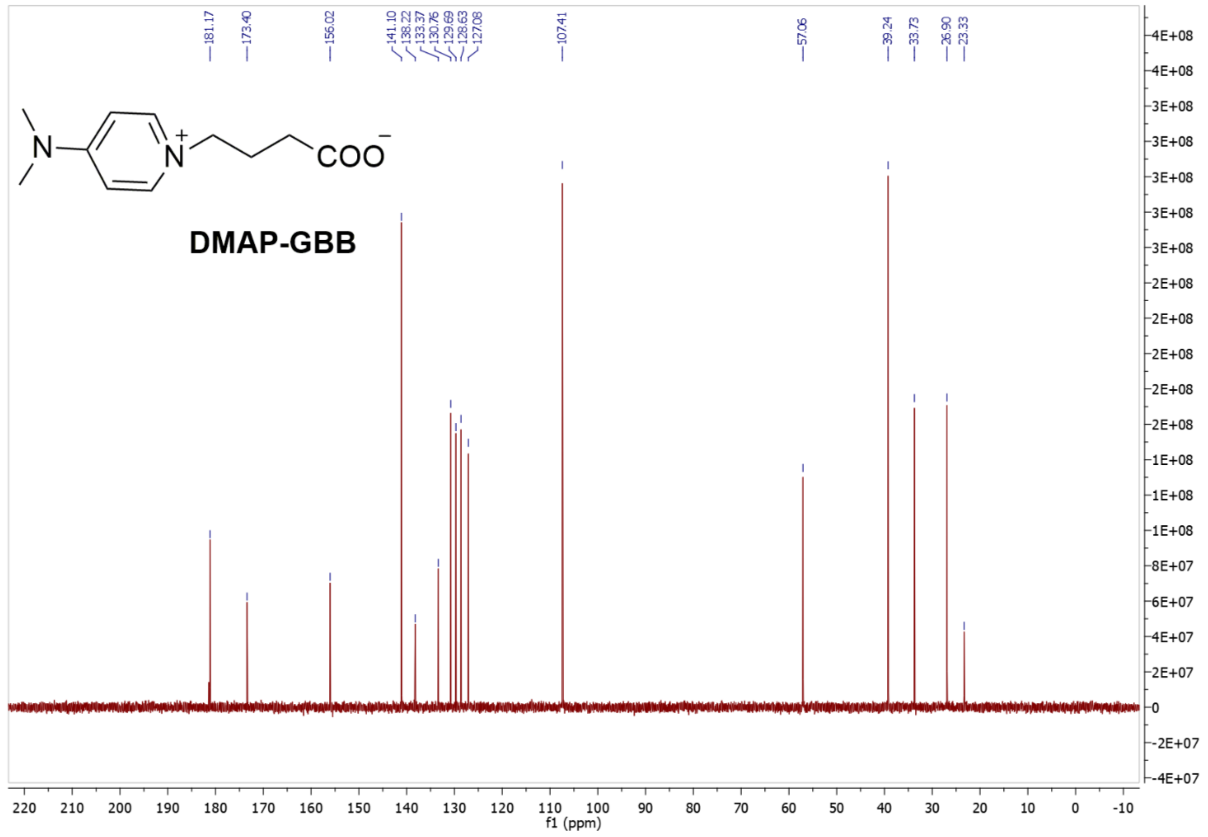

Figure S24. **^{13}C-NMR Spectrum of DMAP-GBB in D_2O-d_2.**

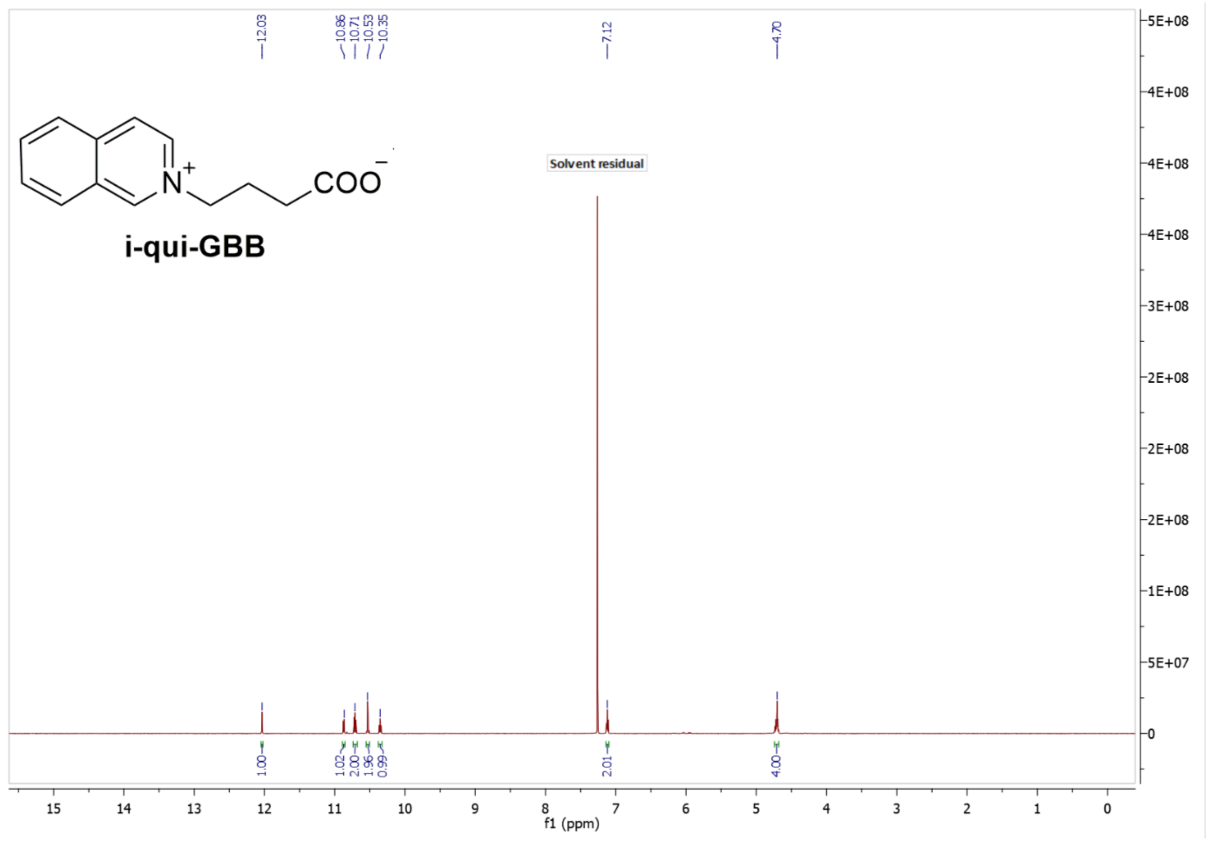

Figure S25. **^{1}H-NMR Spectrum of *i*-qui-GBB in $CDCl_3$-d_1.**

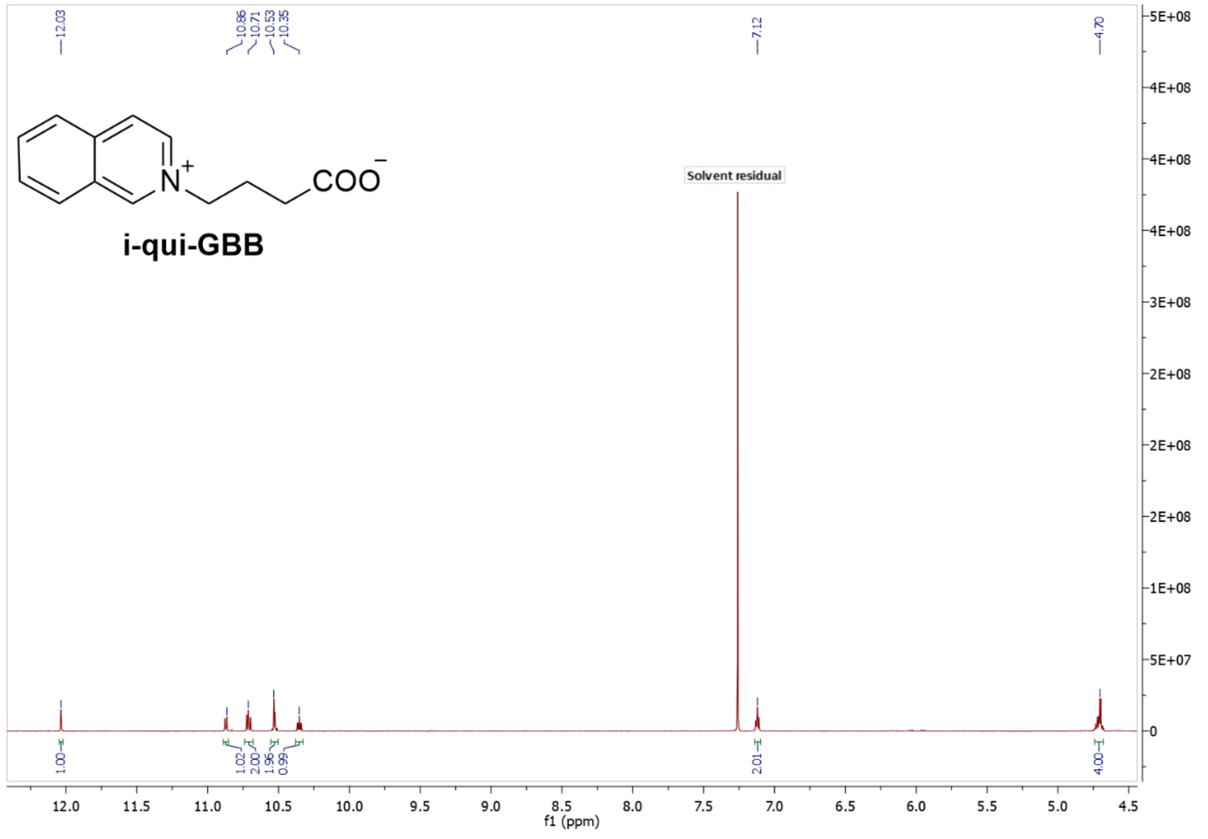

Figure S25a. **^{1}H-NMR Spectrum of *i*-qui-GBB in $CDCl_3$-d_1.**

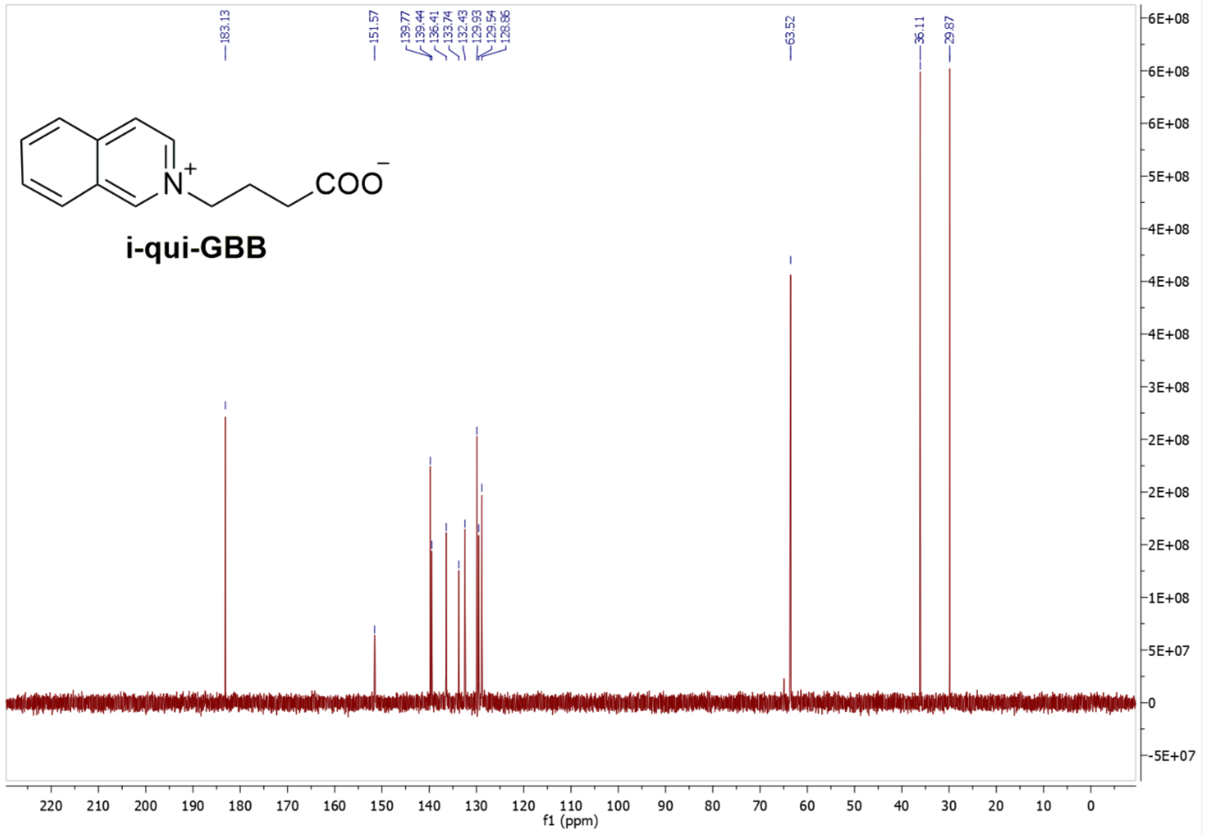

Figure S26. **^{13}C-NMR Spectrum of *i*-qui-GBB in $CDCl_3$-d_1.**
